# iASPP mediates p53 selectivity through a modular mechanism fine-tuning DNA recognition

**DOI:** 10.1073/pnas.1909393116

**Published:** 2019-08-08

**Authors:** Shuo Chen, Jiale Wu, Shan Zhong, Yuntong Li, Ping Zhang, Jingyi Ma, Jingshan Ren, Yun Tan, Yunhao Wang, Kin Fai Au, Christian Siebold, Gareth L. Bond, Zhu Chen, Min Lu, E. Yvonne Jones, Xin Lu

**Affiliations:** ^a^Ludwig Cancer Research, Nuffield Department of Medicine, University of Oxford, Oxford OX3 7DQ, United Kingdom;; ^b^State Key Laboratory of Medical Genomics, Shanghai Institute of Hematology, Rui Jin Hospital affiliated to Shanghai Jiao Tong University School of Medicine, Shanghai 200025, China;; ^c^Division of Structural Biology, Wellcome Centre for Human Genetics, University of Oxford, Oxford OX3 7BN, United Kingdom;; ^d^Department of Internal Medicine, University of Iowa, Iowa City, IA 52242;; ^e^Department of Biostatistics, University of Iowa, Iowa City, IA 52242

**Keywords:** p53, iASPP, crystal structure, target selectivity, HPV E6

## Abstract

The most frequently mutated protein in human cancer is p53, a transcription factor (TF) that regulates myriad genes instrumental in diverse cellular outcomes including growth arrest and cell death. Cell context-dependent p53 modulation is critical for this life-or-death balance, yet remains incompletely understood. Here we identify sequence signatures enriched in genomic p53-binding sites modulated by the transcription cofactor iASPP. Moreover, our p53–iASPP crystal structure reveals that iASPP displaces the p53 L1 loop—which mediates sequence-specific interactions with the signature-corresponding base—without perturbing other DNA-recognizing modules of the p53 DNA-binding domain. A TF commonly uses multiple structural modules to recognize its cognate DNA, and thus this mechanism of a cofactor fine-tuning TF–DNA interactions through targeting a particular module is likely widespread. Previously, all tumor suppressors and oncoproteins that associate with the p53 DNA-binding domain—except the oncogenic E6 from human papillomaviruses (HPVs)—structurally cluster at the DNA-binding site of p53, complicating drug design. By contrast, iASPP inhibits p53 through a distinct surface overlapping the E6 footprint, opening prospects for p53-targeting precision medicine to improve cancer therapy.

## Significance

*TP53*, encoding p53, is the most frequently mutated gene in human cancers. p53 is a transcription factor that suppresses tumors by regulating myriad genes critical for diverse cellular outcomes including growth arrest and death. This study addresses the mechanism by which iASPP, a p53 partner, influences p53 target gene selection. Using next-generation sequencing, we found genes coregulated by iASPP and p53, and characterized their DNA sequence signatures. Our crystal structure of iASPP and p53 reveals that iASPP displaces a loop of p53 that recognizes DNA signatures. iASPP inhibits p53 through a protein surface distinct from other characterized p53 cellular partners but overlapping that targeted by the viral oncoprotein human papillomavirus E6. These findings open prospects for designing p53-targeting anticancer agents.

The recognition of specific DNA sequences by transcription factors (TFs) is instrumental for decoding genomes (1, 2). Beyond the intrinsic TF sequence preferences, TF–DNA interactions are further regulated, in a sequence-specific fashion, through mechanisms such as interplay between TFs (3) and transcription cofactors that modify TFs (4, 5), DNA (6, 7), and histones (8). Intriguingly, a category of transcription cofactors does not appreciably bind DNA and lacks apparent enzymatic activities. Instead, the transcription cofactors directly interact with the DNA-binding domains (DBDs) of TFs and alter TF–DNA binding, thereby endowing partner TFs with transcriptional target gene selectivity (9, 10). Hitherto, the sequence and structural basis for this mode of TF regulation remains poorly characterized.

The apparently 53-kDa tumor suppressor and TF p53 is the most frequently mutated protein in human cancer (11, 12). As a master TF for stress responses, p53 regulates a complex array of genes that can determine diverse cellular outcomes such as growth arrest or death (13–15). How p53 regulates discrete subsets of target genes and why p53 induction leads to cell-cycle arrest in some cell types and apoptosis in others are not completely understood (16).

A common landmark of virtually all p53 target genes is a stretch of specific DNA sequence, termed a response element (RE). These p53 REs share the consensus sequence motif consisting of 2 half sites, each being a 10-base pair (bp) palindrome 5′-PuPuPuC(A/T)(T/A)GPyPyPy-3′ (Pu, purine; Py, pyrimidine), occasionally separated by a short spacer (17, 18). The intrinsic sequence specificity of p53 is principally determined by the multiple DNA-binding modules integrated in its DBD. The p53 DBD has an immunoglobulin (Ig)-like β-sandwich scaffold that presents the so-termed loop–sheet–helix (LSH) motif to fit snugly in the major groove of a DNA double helix and the L3 loop to contact the adjacent minor groove as well as the DNA backbone (19), and a p53 tetramer recognizes the full RE (20). Crystal structures of p53–DNA complexes show that, within the LSH motif responsible for direct base recognition, R280 from the H2 helix makes invariant contacts with the conserved guanine in the REs, whereas K120 from the L1 loop interacts with the neighboring purines (on the opposite strand) in a sequence-dependent manner (19–25). Consistently, the L1 loop has been associated with p53 target selectivity (26, 27). Notably, the acetylation of K120 within L1 further contributes to p53 promoter specificity (5, 28).

The ASPP (apoptosis stimulating protein of p53) family of transcription cofactors represent well-recognized promoter-specific regulators of p53 (14, 29), and exemplars to explore further mechanisms of TF selectivity in general (9, 30). ASPP1 and ASPP2 promote transcriptional activities of p53 specifically on apoptotic genes such as *BAX* and *TP53I3* (PIG3) (31), whereas iASPP is inhibitory (32). Classically, their carboxyl (C)-terminal conserved regions, each comprising ankyrin repeats and a Src homology 3 (SH3) domain, directly bind to p53 DBD (33–35), and iASPP has been shown to interact additionally with p53 regions flanking its DBD (36, 37). Paradoxically, the cocrystal structure of p53–53BP2 (C-terminal ASPP2) demonstrated that p53-stimulating ASPP2 occupies the DNA-binding surface of p53 (31, 34). The detailed mechanism for sequence-specific regulation of p53 by the ASPP family remains obscure. In this study, we focus on the inhibitory iASPP and used RNA sequencing (RNA-seq) combined with chromatin immunoprecipitation followed by sequencing (ChIP-seq) to investigate genome-wide p53 binding and transcriptional activities regulated by iASPP in the HCT 116 colorectal carcinoma cell line, which harbors wild-type p53. This led to the identification of sequence signatures enriched in iASPP-modulated p53 REs and associated target genes. In pursuit of the structural basis of this selective p53 regulation, we solved the crystal structure of a p53–iASPP complex and found that iASPP segregates the L1 loop of p53, which specifies the sequence signatures, from other DNA-binding modules. It does so without blocking the major DNA-binding surface of p53, a unique feature among structurally characterized p53-interacting proteins.

## Results

### Sequence Signatures Enriched in iASPP-Regulated p53 REs.

**Fig. 1.**
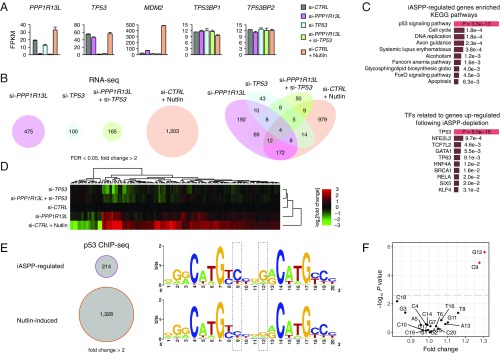
Genomic analysis of gene regulation by iASPP reveals sequence signatures enriched in iASPP-regulated p53 REs. (*A*) Fragments per kilobase of transcript per million mapped reads (FPKM) values from RNA-seq analysis of HCT 116 cells under each experimental condition (see color codes on figure) for *PPP1R13L* (iASPP), *TP53* (p53), *MDM2*, and 2 control genes, *TP53BP1* and *TP53BP2* (ASPP2) that have not been described as p53 transcriptional targets yet encode p53-binding partners. Two batches of RNA-seq data (*n* = 2) were used. Error bars denote standard deviations (SDs). (*B*) The number of DE genes (FDR < 0.05, fold change > 2) under each condition compared to the control siRNA treatment found by RNA-seq (*Left*). Venn diagram analysis (*Right*) of the DE genes. (*C*) Gene set enrichment analysis of iASPP-regulated genes in KEGG pathways (475 genes) (*Top*) and genes up-regulated following iASPP depletion by related TFs (194 genes) (*Bottom*). (*D*) Heat map showing RNA-seq derived gene expression profiles from RNAi experiments (listed *Left*) for 145 p53-regulated and -bound targets identified in this study. FPKM values plus 1 were used for the logarithmic calculations of fold changes. See *SI Appendix*, Fig. S1*B* for the definition of the 145 genes. (*E*) ChIP-seq using a p53 antibody (FL-393) identified 214 elevated p53-binding peaks (fold change > 2) in iASPP-depleted cells versus control siRNA and 1,328 p53-binding peaks (fold change > 2) induced by Nutlin compared to the DMSO control. Sequence logos depicting nucleotide distributions of the 20-base pair consensus p53-binding site based on iASPP-regulated (*Top*) or Nutlin-induced (*Bottom*) response elements (spacers between half sites removed) generated using WebLogo (http://weblogo.berkeley.edu/). Prominent differences of nucleotide distributions at positions 9 and 12 (framed in dashed lines) were observed. C9: 152 out of 208 REs or 73.1% in iASPP-regulated compared to 738 out of 1,290 REs or 57.2% in Nutlin-induced; 1.28-fold, *P* = 1.30 × 10^−5^. G12: 152 REs or 73.1% in iASPP-regulated compared to 722 REs or 56.0% in Nutlin-induced; 1.31-fold, *P* = 2.38 × 10^−6^. Concurrent: 121 out of 208 REs or 58.2% in iASPP-regulated compared to 541 out of 1,290 REs or 41.9% in Nutlin-induced; 1.39-fold, *P* = 1.63 × 10^−5^. Fisher’s exact test was used for the statistical analysis. (*F*) Scatterplot showing the enrichment of each nucleotide of the consensus p53-binding motif on the *x* axis (fold change) and the corresponding *P* value on the *y* axis (−log_10_ scale) found in iASPP-regulated relative to Nutlin-induced p53 ChIP peaks. The fold changes and *P* values were calculated using 2-tailed Fisher’s exact test. The horizontal dashed line represents the Bonferroni-corrected *P* value of 0.05.

To expand knowledge of iASPP-regulated p53 target gene selectivity from a handful of tested promoters to the breadth of the human genome, we initially attempted to generate iASPP-ablated derivatives of wild-type p53-expressing cell lines using CRISPR-Cas9. Unfortunately we were unable to generate such cell lines, presumably due to the inhibition of CRISPR-Cas9 gene editing by hyperactive p53 signaling following iASPP deletion (32, 38, 39). Instead, by RNA interference (RNAi) we transiently depleted iASPP (*PPP1R13L*) in the HCT 116 colorectal carcinoma cell line, which expresses wild-type p53, and analyzed the effects on gene expression with RNA-seq (Fig. 1*A*).

A total of 475 genes were differentially expressed (DE) (false discovery rate [FDR] < 0.05, fold change > 2) in iASPP-depleted cells compared to control cells, comprising 194 up-regulated and 281 down-regulated genes (Fig. 1*B* and *SI Appendix*, Fig. S1*A*). Gene set enrichment analysis of these DE genes showed that iASPP-regulated genes are enriched in the canonical p53 signaling pathway (Kyoto Encyclopedia of Genes and Genomics [KEGG] hsa04115, *P* = 3.5 × 10^−13^) and the transcripts up-regulated by iASPP RNAi are enriched in transcriptional targets of p53 (*P* = 8.0 × 10^−15^) (Fig. 1*C*). As a positive control for p53 activation, we treated HCT 116 cells with Nutlin, a small molecule that stabilizes p53 by blocking MDM2-mediated p53 degradation. In Nutlin-treated cells, 1,203 genes were differentially expressed (Fig. 1 *A* and *B* and *SI Appendix*, Fig. S1*B*). We observed significant albeit relatively weak up-regulation of iASPP by Nutlin (FDR = 0.03, fold change = 1.7) (Fig. 1*A*), consistent with iASPP being a p53 transcriptional target (40) and contributing to p53 negative feedback. The concurrent depletion of iASPP and p53 (*TP53*) resulted in a gene expression profile more closely resembling that of p53 RNAi than iASPP depletion (Fig. 1 *A*, *B*, and *D*). These results suggest that iASPP-mediated gene regulation predominantly acts genetically upstream of the TF p53, which has a low transcriptional activity under steady-state conditions in cancer-derived HCT 116 cells.

To assess genome-wide p53 binding regulated by iASPP, we performed ChIP-seq using an anti-p53 antibody in HCT 116 cells treated with control or iASPP RNAi. A total of 214 p53-binding sites showed elevated signals (fold change > 2) following iASPP depletion (Fig. 1*E*). We used a previously reported position weight matrix algorithm to predict p53 REs within the iASPP-regulated p53 ChIP-seq peaks (41), and generated sequence motifs to analyze nucleotide frequency (highest-scored RE per peak; 208 REs from 214 peaks) (Fig. 1*E*). When the nucleotide distributions of the iASPP-regulated motif were compared to the consensus motif from the Nutlin-induced p53 REs (1,290 REs from 1,328 peaks), we identified clear differences at 2 nucleotide positions (Fig. 1 *E* and *F*). In iASPP-regulated REs, a cytosine (C) was more prevalent at position 9 of a typical 20-bp p53 consensus motif while a guanine (G) was more dominant at position 12 than in Nutlin-induced REs (Fig. 1*E*; C9: *P* = 1.30 × 10^−5^; G12: *P* = 2.38 × 10^−6^; see figure legends for detailed statistics). The concurrent presence of C9 and G12 was enriched in iASPP-regulated compared to Nutlin-induced REs (*P* = 1.63 × 10^−5^). These findings suggest that the presence of C9 and/or G12 in a typical 20-bp p53 motif likely represents the sequence basis for iASPP-regulated p53 binding, which in turn underlies gene regulation by iASPP.

### Identification of iASPP-Regulated p53 Target Genes.

**Fig. 2.**
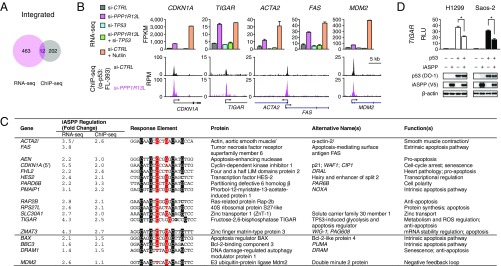
iASPP-regulated p53 target selectivity. (*A*) Intersection of DE genes identified by RNA-seq and genes with differential p53 binding identified by ChIP-seq in iASPP-depleted cells. (*B*) FPKM values from RNA-seq analysis of different cellular conditions (*Top*) and University of California Santa Cruz (UCSC) Genome Browser views of p53 occupancy from ChIP-seq (*Bottom*) for representative p53 targets from the above-mentioned targets coregulated by iASPP, plus *MDM2* as a well-known direct p53 target that did not show substantial coregulation by iASPP. Two batches of RNA-seq data (*n* = 2) were used. Error bars denote SDs. RPM, reads per million; kb, kilobase. (*C*) Thirteen identified iPTGs, together with some representative p53 target genes (below the line), are listed with their observed iASPP-regulated fold changes in RNA levels and p53 ChIP binding as well as identified p53 REs and major associated molecular functions. The signature-corresponding C9 and G12 (highlighted in red) as well as the conserved C4, G7, C14, and C17 (highlighted in black) of a typical 20-bp unsplit p53 consensus motif are indicated and deviations are in bold. (*D*) Luciferase reporter assay of transactivation of p53 response element in *TIGAR* by p53 with cotransfection of iASPP in p53-null cancer cell lines H1299 and Saos-2. RLU, relative luminescence unit (fold changes relative to vector alone are indicated). Three experiments were carried out in triplicate, and data from a representative set are shown. Error bars denote SDs. *P < 0.001 (2-tailed *t* tests). Samples from the triplicates under the same condition were pooled together for Western blotting. Raw data for *D* are available from Mendeley Data at http://dx.doi.org/10.17632/j75wt9b36n.1.

To identify direct, iASPP-regulated p53 target genes (iPTGs), we integrated the RNA-seq and ChIP-seq results and found 13 iPTGs associated with 12 differential p53 ChIP-seq peaks following iASPP depletion (Fig. 2 *A*–*C* and *SI Appendix*, Fig. S2). One of the iASPP-regulated p53 ChIP peaks is shared by the overlapping gene bodies of *ACTA2* and *FAS*, and iASPP depletion promotes the transcription of both genes (Fig. 2*B*). We examined the REs within these 12 iASPP-regulated p53 ChIP-seq peaks and confirmed the concurrent presence of C9 and G12 in 9 out of the 12 p53-binding sites (Fig. 2*C*). We also extended our search in representative p53 target genes and noted that this sequence signature is present in p53 REs responsible for controlling key apoptotic effectors BAX and PUMA (*BBC3*) in addition to FAS and NOXA (*PMAIP1*) identified above (Fig. 2*C*). Conversely, p53 REs for target mediators involved in some other functions, such as DRAM1 (autophagy) and MDM2 (negative feedback) (15), do not bear the full signature (Fig. 2*C*).

The 13 identified iPTGs are involved in diverse biological functions (Fig. 2*C*), yet 9 out of 13 iPTGs have been linked to the regulation of apoptosis, a hallmark of the ASPP family (42). *AEN*, *FAS*, *FHL2* (43, 44), and *PMAIP1* promote p53-induced apoptosis, whereas *CDKN1A* (p21), *RAP2B* (45), and *TIGAR* have prosurvival and antiapoptotic properties (15). *RPS27L* (46) and *ZMAT3* (47) have been reported to promote or inhibit apoptosis in a context-dependent fashion. The remaining 4 iPTGs, *ACTA2* (48), *HES2* (49), *PARD6B* (50), and *SLC30A1* (51), are implicated in other molecular processes including smooth muscle contraction, transcriptional regulation, cell polarity, and zinc transport, respectively.

Apart from *CDKN1A*, iASPP has not been studied in relation to the transcriptional regulation of these iPTGs. Following iASPP depletion, our RNA-seq and ChIP-seq data showed strong up-regulation of transcription and enhanced p53 binding for *TIGAR* (Fig. 2*B*), which modulates metabolism and lowers intracellular reactive oxygen species (ROS) levels in response to mild metabolic stress signals in favor of cell survival (52). We confirmed this result in a luciferase reporter assay using p53-null cell lines H1299 and Saos-2, in which iASPP inhibited p53-mediated transactivation of a *TIGAR* response element (Fig. 2*D*). In contrast, iASPP depletion resulted in only weak induction of *MDM2* (compared to substantial induction in the presence of Nutlin), and *MDM2* had similar p53 ChIP-seq signals in the presence or absence of iASPP (Fig. 2*B*), which is consistent with a previous report (36). This suggests that iASPP does not directly regulate MDM2-based p53 negative feedback. Overall our combined genomic analysis reveals iASPP-mediated, sequence-specific, differential regulation of p53 RE binding and identified a set of previously unrecognized iPTGs.

### Crystal Structure of a p53–iASPP Complex.

**Fig. 3.**
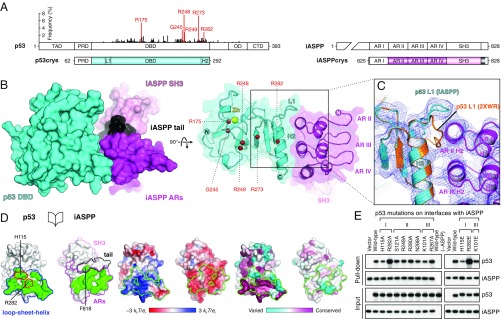
Crystal structure of the p53–iASPP complex. (*A*) Schematic domain structures of human p53 (*Left*) and iASPP (*Right*). The codon distribution of somatic point mutations of p53 derived from cancer patients (*n* = 24,320; International Agency for Research on Cancer [IARC] R18) is plotted above the illustration and the 6 hotspots are labeled in red. The crystallization constructs (p53crys and iASPPcrys) are shown below the full-length proteins with the regions resolved in the crystal structure colored. This color scheme is used in all of the following figures unless otherwise stated. Note that L1 and H2 of p53 are distant in sequence yet structurally close together. AR I–IV, ankyrin repeats I–IV. (*B*) Surface (*Left*) and cartoon (overlaid with near-transparent surface) (*Right*) representations of the interface I-mediated p53–iASPP complex. The p53-bound Zn^2+^ ion is shown as a yellow sphere and the Cα atoms of the p53 mutational hotspots are in red. Domains are colored as in *A*. (*C*) Electron density (2*F*_O_ − *F*_C_ map as blue mesh) at p53–iASPP interface I, contoured at 1.0 σ. An apo p53 DBD structure (orange) with an extended N terminus (PDB entry 2XWR chain B; the search model for molecular replacement) is superposed onto the iASPP-bound p53. (*D*) An open-book view showing interface I (green) with structural units outlined (*Left*). Critical p53 residues for iASPP binding are outlined in red. Solvent-accessible p53 and iASPP surfaces are colored by electrostatic potential (*Middle*) and residue conservation (*Right*) with interface residues outlined in green. (*E*) Binding assay using His-tagged iASPPcrys to pull down full-length p53 and p53 mutants harboring single-point mutations targeting the interfaces with iASPP observed in the crystal lattice. Wild-type (−ASPP) lanes reflect reactions containing wild-type p53 but without the addition of recombinant ASPP polypeptides. Raw data for *E* are available from Mendeley Data at http://dx.doi.org/10.17632/j75wt9b36n.1.

We then set out to understand the structural basis for iASPP-mediated regulation of p53–DNA interactions. The domain organization of a p53 monomer consists of an amino (N)-terminal transactivation domain (TAD), a proline-rich domain (PRD), a central, conserved sequence-specific DBD, an oligomerization domain (OD) that mediates tetramerization, and a carboxyl (C)-terminal regulatory domain (CTD) (Fig. 3*A* and *SI Appendix*, Fig. S3*A*) (21). For crystallographic analysis we used a p53 construct that includes the PRD and DBD (residues 62 to 292) (Fig. 3*A* and *SI Appendix*, Fig. S3*B*) and a C-terminal iASPP construct (residues 625 to 828) (Fig. 3*A* and *SI Appendix*, Figs. S4 *A* and *B* and S5*A*). These constructs enabled crystallization of the purified complex (see *Materials and Methods*). X-ray diffraction data were collected from microcrystals using a synchrotron light source and processed to 4.25-Å resolution, allowing structure determination of the complex by molecular replacement (*Materials and Methods* and *SI Appendix*, Table S1).

Our final model includes p53 residues 91 to 291 with a coordinated zinc^2+^ ion and iASPP residues 657 to 823; the crystallographic asymmetric unit contains 1 such complex (Fig. 3*B*). There was a lack of density for the proline-rich region of p53 (Fig. 3*B* and *SI Appendix*, Fig. S5 *B* and *C*), suggesting it is not critical for the interaction between these truncated protein constructs. The first ankyrin repeat of iASPP was not traceable in the electron density (Fig. 3*A* and *SI Appendix*, Fig. S5*D*). The structures of the individual components observed in the complex are virtually identical to published structures of the p53 DBD fold (a root-mean-square deviation [RMSD] of 0.28 Å for 194 Cα pairs compared to PDB entry 2XWR chain B) and iASPP (an RMSD of 0.57 Å over 166 Cα pairs compared to PDB entry 2VGE), except for the L1 loop of p53 (residues 115 to 120 not matched for the RMSD calculation) (Fig. 3*C* and *SI Appendix*, Fig. S5 *B*–*D*), as discussed below.

The crystal lattice shows 3 intermolecular interfaces between p53 and iASPP (*SI Appendix*, Fig. S6). Interface I has the largest (1,246.5 Å^2^) buried solvent-accessible surface area. Here, iASPP almost exclusively uses a relatively flat surface of its ankyrin stack (composed of residues exposed on the second α-helix of each ankyrin repeat) to interact with the LSH motif of p53, prizing the L1 loop away from its contacts with the S2 β-strand and the H2 α-helix (Fig. 3 *B* and *C* and *SI Appendix*, Fig. S6). iASPP F818, situated in the C-terminal loop (tail) that is trapped between the ankyrin repeats and the SH3 domain provides an auxiliary hydrophobic shield for the interface (Fig. 3 *B* and *D*). Interface I contains both hydrophobic and hydrophilic elements, and the surfaces are complementarily charged (Fig. 3*D*). Furthermore, the interface area on p53 is composed of evolutionarily conserved and varied patches, whereas on the iASPP ankyrin stack the p53-binding surface is less conserved than the regions that maintain the ankyrin-repeat fold (Fig. 3*D*). It is therefore possible that the modulation of the tumor suppressor p53 by iASPP evolved late in vertebrates.

Interface II is the second largest (1,041.6 Å^2^) and is composed predominantly of hydrophilic interactions. At this interface, iASPP employs a different face of its ankyrin stack to engage p53 at a surface that overlaps with its DNA-binding area (*SI Appendix*, Fig. S6). Interface III is the smallest of the 3 (920.6 Å^2^) where the SH3 domain of iASPP contacts the N-terminal loop of p53 DBD, which wraps around the DBD, and the surrounding p53 residues (*SI Appendix*, Fig. S6).

To investigate which p53–iASPP interface(s) observed in the crystal lattice is required for binding, we performed an alanine scan of p53 residues whose side chains are orientated to contact iASPP exclusively at each interface and assayed the binding of corresponding full-length p53 single-point mutants to our iASPP crystallization construct. Where possible, residues with solvent-exposed side chains on the apo structure of p53 DBD that do not display apparent structural roles were chosen. Alanine substitutions of interface I p53 residues H115 and R282 diminished and enhanced p53–iASPP interactions, respectively, whereas mutations of interface II p53 residues S121, R248, R280, and N288 to alanine did not substantially affect the binding (Fig. 3*E*). Interface III mutation p53K101A weakened p53–iASPP binding but p53R267A retained the wild-type level of interaction (Fig. 3*E*). p53 residue H115 is prominently exposed on the DBD surface of p53 in the free or DNA-bound state, but is buried in the interface I-mediated complex with iASPP (Fig. 3*D*). Our structural and mutational data suggest that p53 H115 is a key residue mediating the p53–iASPP interaction. R282 is 1 of the 6 main p53 mutational hotspots and is the only hotspot that is not directly contacting DNA or involved in structuring the DNA-binding L3 loop (Fig. 3*B*). R282 critically maintains the structural integrity of the p53 LSH motif, and the alanine substitution is predicted to disrupt the structural motif and lead to a more flexible L1 loop. We attribute the increased p53R282A–iASPP binding to the destabilization of the local structure and creation of the cleft between the p53 L1 loop and H2 helix for binding iASPP. In this binding assay, we further analyzed charge-swapped p53 H115E, R282E, and K101E. H115E and R282E (interface I) enhanced the respective phenotypes observed with the alanine substitutions, whereas K101E (interface III) had a milder effect on binding (Fig. 3*E*). The results from this binding assay, in conjunction with the structural analysis of the interfaces, led us to assign interface I as the major p53–iASPP interface.

### iASPP Displaces the Signature-Defining L1 Loop of p53.

**Fig. 4.**
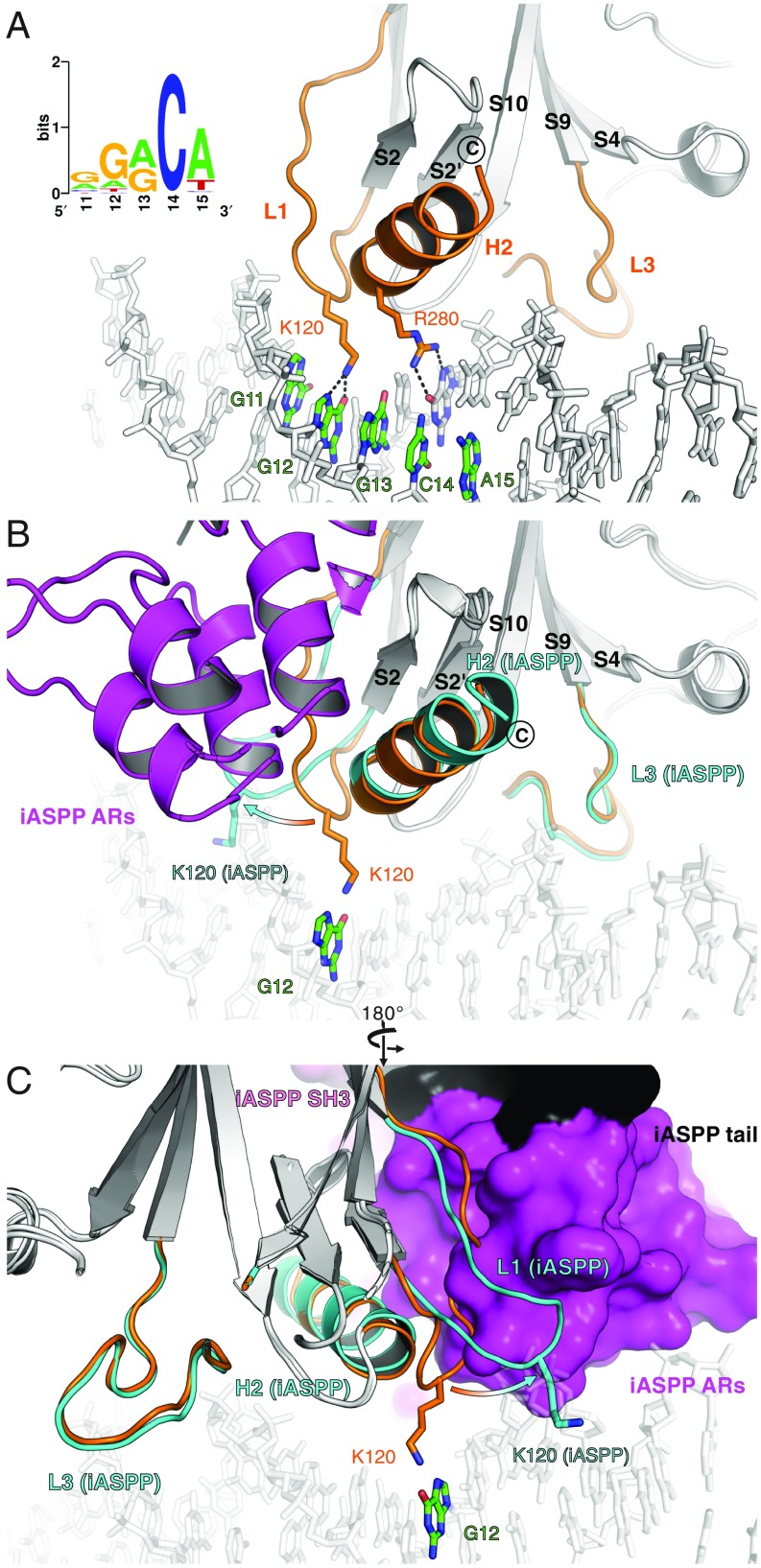
iASPP displaces the L1 loop of p53. (*A*) p53 (cartoon)–DNA (sticks) complex (PDB entry 1TUP; p53 as chain B). The principal DNA-contacting modules of p53 (L1, L3, and H2) are in orange. The side chains of base-recognizing K120 and R280 are illustrated as sticks showing hydrogen bonds with DNA (donor–acceptor distance closer than 3 Å) indicated as dashed lines. A typical pentameric quarter site on the purine-rich strand is highlighted in color. *Inset*, a middle quarter site (positions 11 to 15) from the iASPP-regulated p53 consensus motif. (*B* and *C*) Our iASPP complex superposed onto the classical DNA complex (PDB entry 1TUP) based on p53. iASPP is represented as a cartoon in *B* and solvent-accessible surface in *C*. p53 L1 displacement by iASPP is indicated with an arrow. Orange, in complex with DNA; cyan, with iASPP bound.

We next analyzed the p53–iASPP crystal structure in the context of p53–DNA interactions. Each p53 DBD monomer determines the pentameric consensus DNA duplex motif in the RE, PuPuPuC(A/T) (also called a quarter site). A corresponding middle quarter site from the iASPP-regulated p53 consensus motif determined from our sequence analysis is shown in Fig. 4 *A*, *Inset*. A p53 DBD monomer interacts with this DNA duplex primarily via direct, base-specific major-groove interactions involving the LSH motif. In virtually all p53–DNA structures, R280 from the H2 helix anchors p53 to the cognate DNA duplex through 2 conserved hydrogen bonds to the invariant G on the pyrimidine-rich strand (Fig. 4*A*). In comparison, K120 from the L1 loop prefers a G at the second position of the pentamer (Fig. 4*A*).

We superimposed our iASPP–p53 complex on the classical DNA complex using p53 as the reference (Fig. 4*B*). Overall, iASPP engages p53 on the edge of the DNA-binding site, and does not seem to clash directly with p53-bound DNA (Fig. 4 *B* and *C*). However, the p53 L1 loop is displaced by iASPP such that K120 at its tip is no longer able to make contacts with the base at the second position of a quarter site. The prevalence of a G at this position of a quarter site (G12 as illustrated in Fig. 4 *A*–*C*) corresponds to the sequence signature we identified in iASPP-regulated p53 REs.

In contrast to the displaced L1 loop, the other p53 DNA-binding modules remain well suited to interact with DNA in the iASPP complex. For instance, the H2 helix from the LSH motif and the L3 loop retain essentially the same structures and surface availability in the iASPP complex compared to the DNA-binding structure (Fig. 4 *B* and *C*). In addition, we inspected the surface charge of the p53–iASPP complex along the putative DNA-binding groove (*SI Appendix*, Fig. S7*A*). Although iASPP exhibits a generally negatively charged exterior, the p53–iASPP complex retains a continuous, positively charged surface for binding DNA (*SI Appendix*, Fig. S7*A*).

### iASPP–p53–DNA Assembly Supports the Signature Symmetry.

**Fig. 5.**
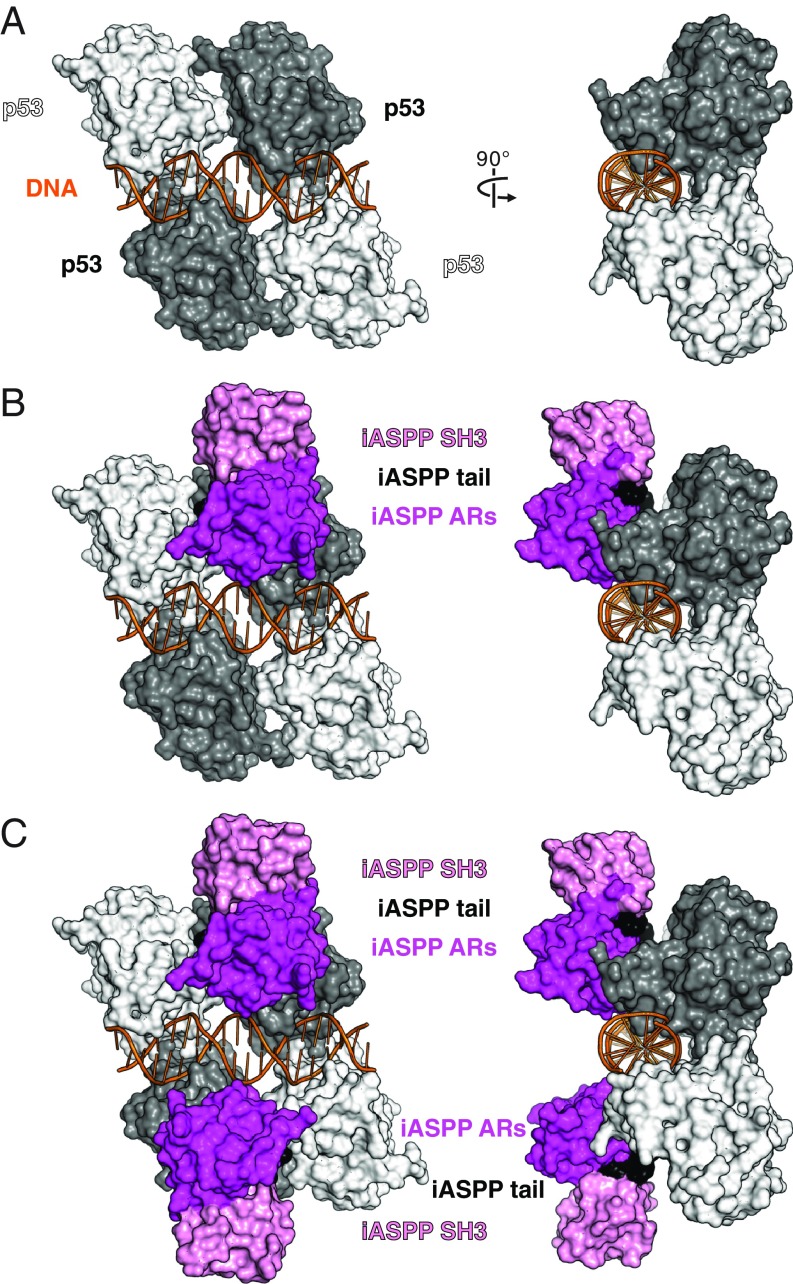
Model of the iASPP–p53–DNA assembly. (*A*) Full consensus site (orange cartoon)-bound tetrameric p53 DBD (surface representation) assembly (PDB entry 3KMD). The middle quarter site-binding p53 DBD monomers are in gray and the outer quarter site-interacting monomers are in white. (*B*) The iASPP complex superposed on the DNA–tetrameric p53 complex based on the superposition of the p53 DBD (1 of the middle p53 DBDs as the reference). (*C*) Model of 2 iASPP molecules docked onto the middle 2 p53 DBD monomers in the DNA–tetrameric p53 complex.

Typically, p53 is held together as a tetramer by its oligomerization domain and recognizes its cognate DNA sequences through its DBD. The consensus motif of p53 REs has a striking symmetry that reflects the dimer-of-dimers architecture of p53 DBDs assembled on cognate DNA sequences, even in the absence of its oligomerization domain (Fig. 5*A*) (22).

When a single p53–iASPP complex is docked onto tetrameric p53–DNA complexes, iASPP does not appear to overlap with any of the symmetry-related p53 DBD molecules (Fig. 5*B* and *SI Appendix*, Fig. S8 *A* and *B*). Superposition of 4 iASPP complexes onto the DNA complexes of p53, on the other hand, would introduce minor clashes between the ankyrin-repeat domains of the adjacent iASPP structures related by the translational symmetry (*SI Appendix*, Figs. S7*B* and S8*C*), although it is possible that a slight architectural adaptation could accommodate a 4:4 iASPP–p53 assembly on an unsplit p53 consensus sequence. Notably, there is no clash between rotational symmetry-related iASPP structures (*SI Appendix*, Figs. S7*B* and S8*C*).

We further noted the structural variations of the L1 loops between the middle and the flanking p53 monomers in the tetrameric p53 assembly on a full consensus site (22, 23) or on the prototypic *CDKN1A* RE (25). K120 at the tip of L1 from each middle p53 DBD is well defined in the major groove to contact DNA but outer L1 loops either become partially disordered (22) or adopt a recessed conformation without direct DNA contacts (23, 25).

These structural observations suggest a functioning model of 2 centrally located iASPP molecules on tetrameric p53–DNA complexes (Fig. 5*C* and *SI Appendix*, Fig. S8*D*) in concordance with the iASPP-regulated p53 RE sequence signatures (C9 [G on the opposite strand] and G12) being symmetrically positioned in the middle p53 quarter sites.

### Distinct p53–ASPP Architectures.

**Fig. 6.**
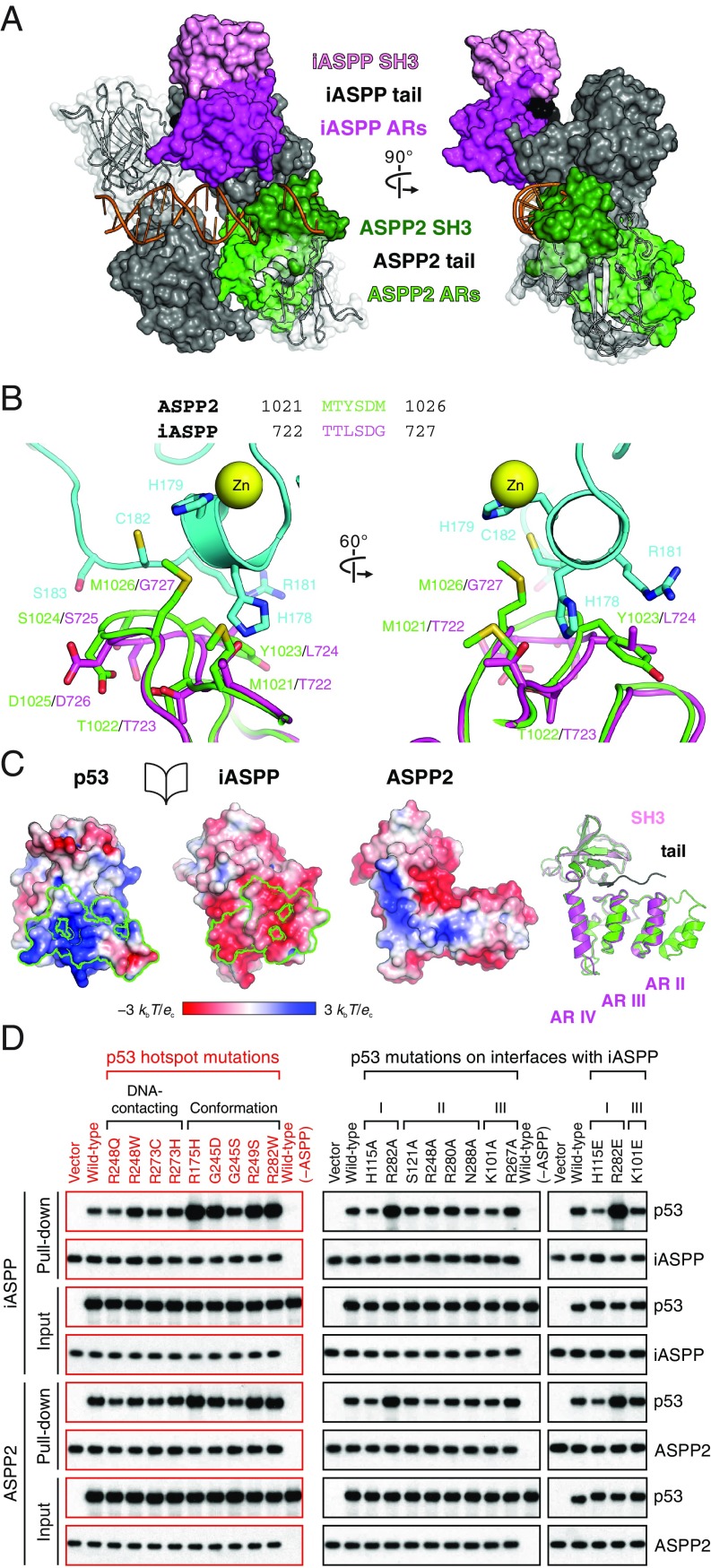
Distinct p53–ASPP architectures. (*A*) iASPP and ASPP2 (ARs in green and SH3 in forest green; PDB entry 1YCS) p53 complexes superposed on the same p53 DBD monomer in a DNA–tetrameric p53 structure (PDB entry 3KMD). (*B*) iASPP as in our p53 complex is superposed onto ASPP2 in its p53 complex. ASPP2–p53 interface residues near the p53 (cyan) Zn (yellow sphere)-binding site are indicated with ASPP2-equivalent iASPP residues illustrated. A structure-based sequence alignment of the ASPP residues is shown (*Top*). (*C*) ASPP2 colored by electrostatic potential (from a perspective corresponding to iASPP illustrated on the *Left*). A superposition of iASPP and ASPP2 (as in their p53 complexes) is shown (*Right*). (*D*) Pull-down assays between full-length p53 (cancer-derived and iASPP-interface mutants) and iASPPcrys or ASPP2crys. Fig. 3*E* is shown here again for comparisons. Raw data for *D* are available from Mendeley Data at http://dx.doi.org/10.17632/j75wt9b36n.1.

Due to the sequence and structural homologies between the C-terminal regions of iASPP and ASPP2 (sequence identity of 54.4% and RMSD of 1.24 Å for 190 Cα pairs), we originally anticipated p53–iASPP interactions to be similar to those described for p53–ASPP2 (34). However, the p53–iASPP structure is distinct from the published p53–ASPP2 complex. ASPP2 binds to p53 DBD at a surface composed predominantly of the L2 and L3 loops, on roughly the opposite side to iASPP (Fig. 6*A*). The superposition of the p53–ASPP2 complex onto a p53–DNA complex shows a major steric clash between the SH3 domain of ASPP2 and the p53-bound DNA (Fig. 6*A*). A substantial steric clash is also observed between the ankyrin repeats of ASPP2 and the rotational symmetry-related p53 DBD monomer required for DNA recognition (Fig. 6*A*).

The area of the interfaces between iASPP and ASPP2 with p53 are similar (∼1,250 Å^2^ vs. ∼1,500 Å^2^, respectively), in line with the comparable p53-binding affinities reported for iASPP and ASPP2 (35). Nevertheless, ASPP2 and iASPP use distinct residues for interaction with p53 (*SI Appendix*, Fig. S4*B*). A close inspection of the p53-contacting residues in iASPP and ASPP2 shows clear differences at the interfaces. At the p53–ASPP2-binding site involving the p53 L2 loop and ASPP2 ankyrin repeat IV (Fig. 6*B*), the bulky side chains of ASPP2 residues (M1021, Y1023, and M1026) that contribute to van der Waals interaction with p53 are divergent in iASPP (replaced by T722, L724, and G727, respectively). In the ASPP2 SH3 domain, L1113 enables optimized binding to p53 but is substituted with a tyrosine residue common for SH3 domains in iASPP (Y814) (34, 36). On the other hand, at interface I of our p53–iASPP complex, the charge complementarity between p53 and iASPP is predicted to be disrupted by the equivalent, positively charged surface on ASPP2 (Fig. 6*C*).

To further examine the differences in the binding of iASPP and ASPP2 to p53, we compared the binding of each to p53 with hotspot mutations found in cancer (Fig. 6*D*). Notably, binding of iASPP to p53 was enhanced by the mutations R175H, G245D, R249S, and R282W (Fig. 6*D*). This is intriguing given that, apart from R282, these residues are not at the iASPP–p53 interface. These mutations have been thought to destabilize p53 DBD structure thereby disrupting its function (19). It has been reported that p53 R282W, in the presence of stabilizing DBD mutations, retains the overall wild-type fold, yet has a disordered L1 loop in a crystal structure (residues 117 to 121) (53). This corresponds to the region that changes conformation upon iASPP binding (Figs. 3*C* and 4 *B* and *C*), rendering the iASPP-binding surfaces more readily accessible. The other mutations that affect p53 DBD conformation (except for G245S which showed more restricted conformational changes in the L3 loop) (53) likely also induce a more open structure in the DBD, particularly in the LSH motif where topologically the N- and C-termini of wild-type p53 DBD meet. Conversely, frequent p53 mutants R248Q, R273C, and R273H, which are believed to largely retain the wild-type DBD fold but are defective in DNA binding (19), bound iASPP similarly to the wild-type tumor suppressor (Fig. 6*D*).

In contrast to the results for iASPP, p53 mutations R248Q and G245S decreased ASPP2–p53 binding, whereas R282W did not substantially affect the interaction (Fig. 6*D*). This is consistent with a previous study looking at the binding between p53 DBD and C-terminal ASPP2 (54). These findings support the model that iASPP and ASPP2 have fundamentally different interactions with p53, and that binding of iASPP is strengthened by mutations that favor a more open structure in the DBD.

### iASPP and HPV E6 Interactions Highlight a Functional p53 Interface.

**Fig. 7.**
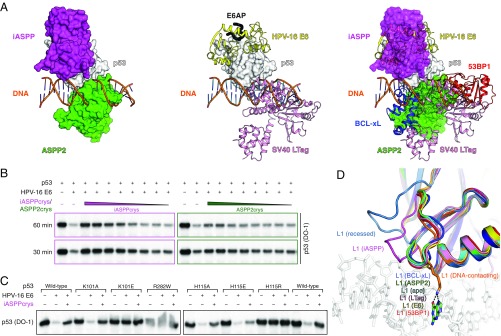
Analysis of p53 DBD complexes. (*A*) Complexes of p53 with iASPP (magenta surface representation) and ASPP2 (green) are superposed on the classical DNA–p53 complex, using p53 DBD (white) as the reference (*Left*). Complexes with viral oncoproteins HPV-16 E6 (E6AP-bound; E6, yellow cartoon; E6AP, black ribbon) and SV40 LTag (pink) were similarly superimposed based on p53 (*Middle*). Other p53 DBD complexes, with 53BP1 (red cartoon) and BCL-xL (blue cartoon) are compared by structural superposition (*Right*). (*B*) In vitro reconstitution of HPV E6-mediated p53 degradation, in the presence of a gradient of concentrations of iASPPcrys or ASPP2crys (same gradient of molar concentration for iASPPcrys and ASPP2crys), tested at 60 min and 30 min. (*C*) Structure-guided p53 point mutations at the interface with E6 and iASPP are tested in the degradation assay at 60 min. (*D*) Superposition of the apo p53 DBD and DBD structures from macromolecular complexes, focusing on the LSH motif. Colors for each complex are indicated. The side chain of p53 K120 from the DNA complex (PDB entry 1TUP chain B) is shown as orange sticks, and DNA from the complex is indicated. Note that the outer p53 monomers in the tetrameric p53 assembly on the major *CDKN1A* RE (PDB entry 3TS8 chain A illustrated) adopt a recessed L1 conformation.

The p53 DBD is not only responsible for sequence-specific interaction with cognate DNA, but is also associated with cellular proteins and targeted by viral oncoproteins. Previously all endogenous partners structurally characterized as p53 DBD complexes, including ASPP2 (34), 53BP1 (55), and BCL-xL (56), cluster at the p53 DNA-binding surface where SV40 LTag interaction maps (57) (Fig. 7*A*). A recent crystal structure determination showed that the HPV oncoprotein E6, in complex with an E6-associated protein (E6AP) peptide, interacts with the N-terminal arm and the LSH motif of p53 DBD (58). This HPV E6-binding site substantially overlaps where iASPP binds in our p53–iASPP structure, and there is considerable steric hindrance between iASPP and E6AP-bound E6 when the complexes are superimposed, based on p53 (Fig. 7*A*). Our structural analysis led us to hypothesize that iASPP may compete with E6 to stabilize p53.

Consistent with these structural observations, in a classical HPV E6-mediated p53 degradation assay, iASPP inhibited p53 degradation induced by the HPV-16 E6 oncoprotein in a dose-dependent manner (Fig. 7*B*). In comparison, purified C-terminal ASPP2 only mildly reduced the degradation of p53 at the highest concentrations administrated (Fig. 7*B*). We further tested single-point mutations of solvent-exposed residues at relevant p53 surfaces and found that the iASPP binding-defective p53 H115A and H115E mutants were as susceptible to HPV-16 E6-mediated degradation as the wild type but the degradation could no longer be efficiently blocked by iASPP (Fig. 7*C*). In line with a previous report (58), the alanine substitutions of p53 residues K101 and H115, which mediate p53–HPV-16 E6 interaction, do not substantially affect p53 degradation induced by HPV-16 E6. However, p53 K101E and H115R mutants are resistant to HPV-16 E6-mediated degradation (Fig. 7*C*).

Notwithstanding their overlapping binding surfaces on p53 DBD, iASPP and HPV E6 adopt distinct molecular mechanisms to inhibit p53 activities. Our p53–iASPP complex reveals that iASPP packs against the H2 helix of p53 DBD and displaces p53 L1 loop from its DNA-binding position, reminiscent of a recessed L1 conformation (25) (Fig. 7*D*), but does not impact DNA binding by other p53 DNA-recognizing modules, which we deem responsible for specific, iASPP-regulated changes in the p53 RE motif characterized in our genomic analysis. These observations are in line with a biological role of iASPP in fine-tuning p53 genomic binding and transcriptional output as a means of p53 regulation. On the other hand, high-risk type HPV E6 binding does not sterically interfere with DNA interaction or introduce any appreciable structural changes to p53 DNA-binding modules. The L1 loop of p53 in complex with E6 aligns well with apo and DNA-binding L1 structures and is favorably positioned to interact with DNA. In fact, the p53 L1 appears somewhat restricted in this position as a result of E6 interaction. Instead of steric hindrance or allosteric regulation, HPV E6 recruits E6AP to ubiquitinate p53 and in turn mediate p53 degradation, thereby attaining a more thorough inhibition for viral reproduction. Thus, although iASPP and E6 share an interaction surface, iASPP has a unique mode of p53 regulation.

## Discussion

Our structure of the p53 DBD in complex with iASPP exemplifies a mechanism through which direct interactions with partner proteins impart DNA-binding refinement and target gene selectivity to transcription factors. The accomplishment of binding affinity and sequence specificity across a genome entails multiple DNA-interacting structural modules of a TF. In relation to protein–DNA interactions, p53 resembles most gene regulatory proteins in that it uses an α-helix (the C-terminal H2 in the DBD) to contact the major groove of cognate DNA (19). In conjunction with the L3 loop, H2 interactions define p53 binding to the conserved core of its cognate DNA half site. Notably, the L1 loop of p53 packing against H2 makes further, sequence-specific interactions with more variable bases flanking the core motif in the major groove, corresponding to the sequence signatures enriched in iASPP-regulated p53 response elements. A similar helix–loop arrangement has been described for the GATA-1–DNA complex (59). Our genomic and structural analyses advocate that iASPP achieves differential p53 inhibition by modulating the peripheral contacts made by p53 L1 without disrupting the anchoring p53–RE interactions mediated by its H2 and L3 modules. TF regulatory proteins could act similarly to iASPP to modulate TF target selectivity. Furthermore, the displacement of p53 L1 by iASPP could potentially affect p53–Tip60 interactions and Tip60-mediated acetylation of L1 residue K120. Since K120 acetylation is critical for p53-dependent apoptosis but dispensable for growth arrest (5), this mechanism could contribute to the modulation of p53 target gene selectivity by iASPP. Beyond the modulation of p53–DNA interactions, iASPP binding might influence p53 interactions with the general transcriptional machinery, for example the RPB1 and RPB2 subunits (60) of the RNA polymerase II, which could contribute to the differential p53 regulation by iASPP observed in our genomic analysis.

Our genome-wide surveys provide a more complete landscape of iASPP-mediated p53 regulation. We identified iPTGs previously not associated with iASPP, for instance *TIGAR*, an antiapoptosis and prosurvival gene, involved in metabolism and ROS control. The majority of the identified iPTGs participate in p53-mediated cellular life-or-death decisions, in line with an apoptosis-related function of iASPP. Of note, several iPTGs, such as *ACTA2*, *FHL2*, *PARD6B*, and *SLC30A1*, may be linked to iASPP-related phenotypes such as sudden cardiac death and dilated cardiomyopathy (61, 62).

The distinct p53 interfaces of the ASPP family members, iASPP and ASPP2, are unexpected and the observed differential interactions offer a fresh perspective on the mechanisms governing the opposing biological functions of the ASPP molecules in regulating p53 and its family members p63 and p73 in development, tissue homeostasis, and cancer. In accord with the ASPP2–p53 structural study (34), the C-terminal region of ASPP2 competes with DNA for p53 binding in an in vitro assay (54) and inhibits p53 transcriptional activity in cells (31). Full-length ASPP2, on the other hand, is able to stimulate the target selective transcriptional activity of p53 on genes such as *BAX* (31), indicating this stimulatory activity of ASPP2 requires the rest of the protein. It remains obscure how full-length ASPP2 selectively stimulates the transcriptional activities of p53. In light of the p53–iASPP structure shown here, it is possible that full-length ASPP2 may compete with iASPP to bind p53 via a similar L1 loop-containing interface and/or to form ASPP2–p53–DNA ternary complexes in cells to stimulate p53 target-selective transcription. Interestingly, previous nuclear magnetic resonance (NMR) characterization of p53–ASPP2 interactions in solution showed that the binding of C-terminal ASPP2 induces structural changes in L1 and H2 of p53 in addition to the binding surface described in the early crystal structure (34, 54), suggesting the possibility that our observed p53–iASPP interface is also relevant to p53–ASPP2 interactions as a secondary binding site. Furthermore, the linker between p53 DBD and OD was shown to contribute to binding the iASPP SH3 domain (37), which is available for interaction in our p53 DBD complex. These discrete p53–iASPP interactions may combine to provide affinity and specificity required for iASPP-mediated p53 regulation.

Mapping cancer-derived iASPP mutations in relation to our crystal structure of the p53 complex did not show a clear pattern (*SI Appendix*, Fig. S7*C*). Indeed, there is growing evidence that overexpression, rather than mutation, of iASPP conveys its oncogenic properties. In addition to our previous characterization in p53 wild-type breast cancer (32), elevated iASPP levels have recently been reported in multiple human cancers, including bladder cancer (63), non-small-cell lung cancer (64), ovarian clear cell carcinoma (65), colorectal cancer (66), and particularly in acute leukemia where p53 mutations are relatively rare (67, 68).

A crucial advance from this study is the identification of a second molecular interaction surface on p53 DBD that holds promise for targeted cancer therapies. ASPP2 and iASPP mirror the p53 interactions of 2 of the best-characterized p53-hijacking viral oncoproteins, SV40 LTag and HPV E6, respectively. The ASPP2 and SV40 LTag-binding site, involving the L3 loop of p53, represents a classic p53 interaction hub that contacts both DNA and other proteins such as 53BP1 and BCL-xL. This p53 surface is also where most cancer-derived mutations map and 5 out of 6 p53 mutational hotspots locate here. However, it presents a formidable challenge for therapeutic targeting because this surface is required for DNA interaction. On the other hand, the iASPP site, which is targeted by HPV E6, does not directly engage DNA. Hotspot R282 stabilizes p53 L1 at this surface (53), and our finding of increased iASPP binding to tumor-derived p53 R282W could contribute to the oncogenic properties of this mutation. The intrinsic flexibility of iASPP-interacting L1 could be modulated by engineered mutations (69), and has been proposed for reactivation of p53 mutants (70). In agreement with this hypothesis, an ASPP2-derived peptide that binds L1 and H2 of p53, has been shown previously to stabilize mutant forms of p53 and rescue p53 functions in cells (71, 72). Our results in conjunction with previous studies provide a structural and functional framework for designing p53-targeting anticancer agents to achieve dissociation of oncoproteins inhibiting wild-type p53 as well as rescue of p53 mutations.

## Materials and Methods

HCT 116 cells were treated with siRNA molecules (Dharmacon) and used to perform the RNA-seq and the ChIP-seq (p53 antibody FL-393, Santa Cruz Biotechnology sc-6243) experiments. The sequencing data were analyzed with standard bioinformatics pipelines. p53crys (human p53 [UniProt accession code P04637] residues 62 to 292) and iASPPcrys (human iASPP [UniProt Q8WUF5] residues 625 to 828) were expressed in *Escherichia coli* and purified to homogeneity. The p53–iASPP complex was prepared as an equimolar mixture of the purified proteins before size exclusion chromatography. The peak fractions were combined and concentrated for crystallization trials. Diffraction-quality crystals were optimized in 18% (wt/vol) polyethylene glycol 3350, 0.18 M trisodium citrate. Crystallographic data were collected and processed, and the structure was determined and analyzed, essentially as described previously (73). A detailed description of the materials and methods used in this study is provided in *SI Appendix*, *Supplementary Materials and Methods*. Raw data files as noted in the legends of Figs. 2, 3, and 6 are available from Mendeley Data at http://dx.doi.org/10.17632/j75wt9b36n.1.

## Supplementary Material

Supplementary File
