# Supplementary material for: iASPP mediates p53 selectivity through a modular mechanism fine-tuning DNA recognition
Source: Proc Natl Acad Sci U S A. 2019 Aug 8;116(35):17470–9. doi: 10.1073/pnas.1909393116 (PMC6717262; doi:10.1073/pnas.1909393116)
Supplement: Supplementary File [file pnas.1909393116.sapp.pdf]

PNAS
www.pnas.org

Supplementary Information for

iASPP mediates p53 selectivity through a modular mechanism fine-tuning DNA recognition

Shuo Chen[a], Jiale Wu[b], Shan Zhong[a], Yuntong Li[b], Ping Zhang[a], Jingyi Ma[a], Jingshan Ren[c], Yun Tan[b], Yunhao Wang[d], Kin Fai Au[d,e], Christian Siebold[c], Gareth L. Bond[a], Zhu Chen[b,1], Min Lu[b,1], E. Yvonne Jones[c,1], and Xin Lu[a,1]

[a]Ludwig Cancer Research, Nuffield Department of Medicine, University of Oxford, Oxford OX3 7DQ, UK
[b]State Key Laboratory of Medical Genomics, Shanghai Institute of Hematology, Rui Jin Hospital affiliated to Shanghai Jiao Tong University School of Medicine, Shanghai 200025, China
[c]Division of Structural Biology, Wellcome Centre for Human Genetics, University of Oxford, Oxford OX3 7BN, UK
[d]Department of Internal Medicine, University of Iowa, Iowa City, Iowa 52242, USA
[e]Department of Biostatistics, University of Iowa, Iowa City, Iowa 52242, USA

[1]To whom correspondence should be addressed. Email: zchen@stn.sh.cn, min.lu@shsmu.edu.cn, yvonne@strubi.ox.ac.uk, or xin.lu@ludwig.ox.ac.uk.

This PDF file includes:

Supplementary Materials and Methods
Figs. S1 to S8
Table S1
References for SI reference citations

www.pnas.org/cgi/doi/10.1073/pnas.1909393116

Supplementary Materials and Methods

Cell Lines and Cell Culture. HCT 116, H1299, and Saos-2 cell lines derived from the American Type Culture Collection (ATCC) were cultured in Dulbecco's Modified Eagle Medium (DMEM; Gibco) supplemented with 10% (v/v) fetal bovine serum (FBS; Gibco), glutamine, and appropriate antibiotics. Cell cultures were maintained in a 5% CO2 humidified incubator at 37°C.

RNA interference (RNAi). Reverse transfection of HCT 116 cells with siRNA targeting *PPP1R13L* (Dharmacon MU-003815-01-0002) or *TP53* (Dharmacon MQ-003329-03-0002), or siGENOME Non-Targeting siRNA Pool #1 (Dharmacon D-001206-13-50) was performed using DharmaFECT 1 Transfection Reagent (Dharmacon T-2001-02) following the manufacturer's instructions. 3 days post-transfection, cells were treated with Nutlin [(±)-Nutlin-3; Cayman Chemical 10004372] overnight. 3.5 days post-transfection, cells were harvested for evaluation of protein depletion by western blotting, and used for either RNA sequencing (RNA-seq) or chromatin immunoprecipitation (ChIP) followed by sequencing (ChIP-seq) analysis.

RNA sequencing (RNA-seq). RNA was isolated using the AllPrep DNA/RNA Mini Kit (Qiagen 80204) from lysed cells, and mRNA was purified using the Dynabeads mRNA DIRECT Purification Kit (Invitrogen 61011) before being converted to cDNA using the SuperScript II Reverse Transcriptase (Invitrogen 18064014). Sequencing libraries were prepared using the TruSeq RNA Library Prep Kit v2 (Illumina RS-122-2001) and paired-end sequencing was conducted on the Illumina MiSeq platform according to the manufacturer's instructions. The quality of the sequencing reads generated was evaluated by FastQC (version 0.11.3; http://www.bioinformatics.babraham.ac.uk/projects/fastqc/), and the adaptor sequences were trimmed by cutadapt (version 1.8.1) (1). Processed reads were aligned to the hg19 reference human genome using HISAT2 (version 2.0.0-beta) (2) with default parameters. Employing the RefSeq annotation library, Cufflinks (version 2.2.1) (3, 4) with default parameters was used to quantify and normalize gene expression, and to perform differential gene expression analysis [the cutoff significance statistics was false discovery rate (FDR) $q < 0.05$ (corresponding to $P < 0.0127$) and we used a further criterion of gene expression level fold change > 2]. We generated 10 million sequencing reads per sample in the pilot study and confirmed the results by repeating the experiments at the sequencing depth of 50 million reads per sample. The statistical analysis was carried out using these two batches of data. Gene set enrichment analysis was carried out using Enrichr (5).

Chromatin Immunoprecipitation (ChIP) Followed by sequencing (ChIP-seq). ChIP was performed using the ChIP-IT High Sensitivity (HS) Kit (Active Motif 53040) according to the manufacturer's recommended protocol with the following modifications as described. Cells were cross-linked with 1% formaldehyde in DMEM culture medium for 10 min at room temperature, and quenched in 0.125 M glycine for 5 min. Cells were then washed twice with phosphate-buffered saline (PBS) followed by 3 times with lysis buffer [10 mM Tris-HCl (pH 7.5), 10 mM NaCl, 3 mM $MgCl_2$, 0.5% NP-40, 1 mM PMSF], and resuspended in ChIP Buffer (Active Motif 37516) supplemented with 1 mM PMSF and a proteinase inhibitor cocktail (Sigma-Aldrich P2714-1BTL). Chromatin was sheared by sonication (30 s alternated with 30-s cooling for 25 cycles) using a Bioruptor Pico ultrasonicator (Diagenode). Solubilized chromatin was used for immunoprecipitation by incubation overnight at 4°C with 5 μg of the polyclonal antibody FL-393 against human p53 (Santa Cruz Biotechnology sc-6243). The immunoprecipitation reactions were transferred to Protein G Agarose Columns (Active Motif 53039) and incubated for 2 h at 4°C. Then, each column was washed 4 times with 900 μl Wash Buffer AM1. The ChIPed samples were eluted using 100 μl Elution Buffer AM4 at 37°C. Cross-linking was reversed by adding 5 μl Proteinase K (New England Biolabs P8107S) and incubated overnight at 65°C. Input and ChIPed DNA

were purified using the DNA purification columns. ChIP-seq libraries were prepared using MicroPlex Library Preparation Kit v2 (Diagenode C05010014) and the Agencourt AMPure XP system (Beckman Coulter A63880) was used to select 350–550 bp libraries. Sequencing was carried out on the Illumina MiSeq and HiSeq platforms.

All ChIP-seq reads were aligned to the hg38 reference human genome by Bowtie 2 (version 2.2.9) (6); reads mapping to multiple genomic locations were removed. Enriched p53 occupancies relative to a background input were called using MACS (version 1.4) (7) with default settings. Genomic regions with a P value $< 1 \times 10^{-10}$ were retained and these enriched regions were annotated using the annotatePeaks package from the HOMER suite (8). The read counts within 500 bases of each peak summit were extracted using the HOMER suite and used for generating the heat maps of p53 binding. The makeUCSCfile package from the HOMER suite was used to generate bigWig and bedGraph files, and the bedGraph files were uploaded to the UCSC Genome Browser to generate the genomic view of p53 binding signals. A fold change of read counts within the peak region > 2 was deemed significantly different p53 binding. We have carried out the ChIP-seq analysis of iASPP-depleted HCT 116 cells versus control RNAi-treated sample twice, and the experiment that gave better p53 ChIP-seq signals was presented although the fold changes of read counts within the peak regions of interest were similar.

Identification of Putative p53 Response Elements Using a Position Weight Matrix. We used a position weight matrix (PWM) for p53 response elements (REs) described previously (9), which is based on 228 experimentally-established functional p53 REs. Briefly, a PWM scoring model for the p53 REs was constructed to estimate the binding strengths of protein–DNA interactions (10). PWM scores were then calculated and assigned to the p53 REs that overlay the p53 ChIP-seq peaks. The p53 REs with the highest PWM score for each ChIP-seq peak were selected.

Transactivation Assays. H1299 and Saos-2 cells were plated onto 24-well plates (5×10^4 cells in 0.5 ml per well) and transfected on the following day with a total of 210 ng plasmid per well (100 ng reporter plasmid, 10 ng pcDNA3-p53, 100 ng pcDNA3-iASPP or pcDNA3-HPV11E6, and balanced with pcDNA3 as necessary) using FuGENE 6 (Promega E2692). 24 h post-transfection, firefly luciferase activities were measured using the Dual-Luciferase Reporter Assay System (Promega E1980). We observed transactivation of our *Renilla* luciferase control (pRL-TK) by p53 co-transfection and thus excluded the use of pRL-TK. The relative efficiency of transfection was assessed by comparing protein levels.

Cloning, Expression and Purification of Recombinant Proteins. Human iASPP and ASPP2 C-terminal regions (iASPPcrys: UniProt accession code Q8WUF5-1 residues 625–828; ASPP2crys: UniProt accession code Q13625-1 residues 889–1128) were cloned into an in-house derivative of the pET-22b(+) vector (Novagen 69744-3), pET-22b(+)-Avi, for recombinant protein expression (referred to as iASPPcrys and ASPP2crys) using standard molecular cloning techniques. The expression construct of human p53 DNA-binding domain (DBD) plus its preceding proline-rich domain (PRD) (referred to as p53crys; UniProt accession code P04637-1 residues 62–292) was kindly gifted by Doctor Ross Robinson.

Recombinant proteins were expressed in *Escherichia coli* BL21(DE3) cells (Agilent Technologies 200131) grown in lysogeny broth. Protein expression was induced by isopropyl β-D-1-thiogalactopyranoside (IPTG; 0.5 mM final concentration) when the bacterial culture reached an optical density of 0.6 at 600 nm, and subsequently incubated overnight (typically for 20 h) at 18°C. Induced cells were harvested by centrifugation, and either lysed for protein purification straightaway or frozen at −80°C for subsequent protein purification. Zinc sulphate (0.1 mM final concentration) was supplemented to the p53crys culture at the time of induction.

For the purification of iASPPcrys and ASPP2crys (C-terminal His_6-tagged), cells were resuspended in 2 × PBS supplemented with 500 mM NaCl and a protease inhibitor cocktail (Sigma-

Aldrich P8849) and lysed by sonication. The lysate was clarified by centrifugation and filtration before capture of the recombinant proteins by immobilized metal ion affinity chromatography using HisTrap HP columns (GE Healthcare 17524802). The recombinant proteins were further purified with ion exchange chromatography using HiTrap Q HP columns (GE Healthcare 17115401) and finally polished by size exclusion chromatography in 10 mM HEPES-Na (pH 7.4), 150 mM NaCl. For p53crys, cell lysate was similarly prepared before capture of the recombinant protein by ion exchange chromatography using HiTrap SP HP columns (GE Healthcare 17115201). The recombinant protein was further purified with heparin-affinity chromatography using HiTrap Heparin HP columns (GE Healthcare 17040703) and finally polished by size exclusion chromatography in 10 mM HEPES-Na (pH 7.4), 150 mM NaCl.

Crystallization, Data Collection and Structure Determination. The complex of p53crys and iASPPcrys was prepared as an equimolar mixture of the purified recombinant proteins before being subjected to size exclusion chromatography in 10 mM HEPES-Na (pH 7.4), 150 mM NaCl. The peak fractions were combined and concentrated to ~8 mg ml^{-1} for crystallization trials in sitting nanodrops (11). Initial crystals grew at 21°C in 20% (w/v) polyethylene glycol 3350, 0.2 M tri-sodium citrate; optimization yielded slightly larger but still very small (roughly 20 μm × 20 μm × 10 μm) crystals in 18% (w/v) polyethylene glycol 3350, 0.18 M tri-sodium citrate.

Crystals were briefly immersed in mother liquor supplemented with 25% ethylene glycol before flash freezing in liquid nitrogen. X-ray diffraction data were collected at 100 K using a microfocus beam (a cross section of 10 μm × 10 μm) and the PILATUS 6M detector on beamline I24 of the Diamond Light Source.

X-ray diffraction data were indexed, integrated, and scaled with the HKL2000 package (12). The structure of the p53–iASPP complex was solved by molecular replacement using the structures of the apo p53 DBD (with an extended N terminus; PDB accession code 2XWR chain B) and the C-terminal region of iASPP (PDB accession code 2VGE) as search models successively in Phaser (13). The structure was iteratively built manually in Coot (14), refined in Refmac (15) using the high-resolution search models as reference structures, and validated with MolProbity (16). The first ankyrin repeat of iASPP was not traceable in the electron density. There was also lack of density for p53 PRD. The Ramachandran plot of the final structure shows 97.25% and 0.27% of its residues to be in the favored and disallowed regions, respectively. The structural analysis was carried out as described previously (17). Structural alignments were performed using PDBeFold (18) to calculate root-mean-square deviation values. Macromolecular interfaces were analyzed using PDBePISA (19). Electrostatic potential was calculated using APBS (20) and residue conservation mapped onto the structure with the ConSurf server (21). Structural figures were generated using PyMOL (Schrödinger).

Site-Directed Mutagenesis. Site-directed mutagenesis was carried out using standard molecular cloning techniques and all mutants were verified by sequencing.

Histidine-Tag Pull-Down Assay. Wild-type and mutant p53 proteins were in vitro translated using the TNT T7 Quick Coupled Transcription/Translation System (Promega L1170). Reaction mixtures containing in vitro translated p53 proteins (10 μl each) diluted in 20 mM Tris-Cl (pH 8.0), 150 mM NaCl, 1% (v/v) IGEPAL CA-630 (Sigma I8896) and 20 mM imidazole (lysis buffer; 0.5 ml per sample) were incubated with Ni-NTA Agarose (QIAGEN 30210) pre-immobilized with purified iASPP or ASPP2 (iASPPcrys and ASPP2crys respectively; 5 μg immobilized on 10 μl of resin per sample) for 2 h at 4°C. The beads were washed with the lysis buffer for 3 times before the bound proteins were eluted with NuPAGE LDS Sample Buffer (Life Technologies NP0008; 20 μl 2 × per sample) supplemented with β-mercaptoethanol (200 mM) and subject to western blotting. Nitrocellulose membranes were probed with the DO-1 antibody (produced in-

house) or the Penta·His Antibody (QIAGEN 34660). Horseradish peroxidase (HRP)-conjugated secondary antibodies were used.

p53 Degradation Assays. HPV E6 and p53 proteins were in vitro translated using the TNT T7 Quick Coupled Transcription/Translation System (Promega L1170). The HPV E6-mediated degradation of p53 was typically assayed at 26°C for 1 h unless otherwise indicated in 30-µl volumes incorporating 2 µl of translated p53 and 4 µl of translated E6 in 25 mM Tris-Cl (pH 7.4), 100 mM NaCl, and 3 mM dithiothreitol (DTT) for each reaction. Rabbit reticulocyte lysate not programed with exogenous plasmid DNA was used to bring the total volume of lysate in a reaction to 10 µl. Purified recombinant ASPP constructs (2 µl each of highest tested concentration of ~400 µM) or the size exclusion chromatography buffer [10 mM HEPES-Na (pH 7.4), 150 mM NaCl] were added, as indicated, to the degradation assay mixtures to assess their impact on HPV E6-mediated p53 degradation. The reactions were terminated by the addition of NuPAGE LDS Sample Buffer supplemented with β-mercaptoethanol followed by boiling. The reaction mixtures were then subject to SDS PAGE and the proteins of interest were visualized by western blotting.

Materials and Data Availability. All sequencing data have been deposited in the Gene Expression Omnibus (GEO) database under accession codes GSE111798 (RNA-seq) and GSE113338 (ChIP-seq). Atomic coordinates and structure factors for the p53–iASPP crystal structure have been deposited in the Worldwide Protein Data Bank (wwPDB) under accession code 6RZ3. Raw data for western blotting and luciferase reporter assays used in Figs. 2, 3, and 6 are available from Mendeley Data (http://dx.doi.org/10.17632/j75wt9b36n.1). All other data and materials are available from the corresponding authors upon request.

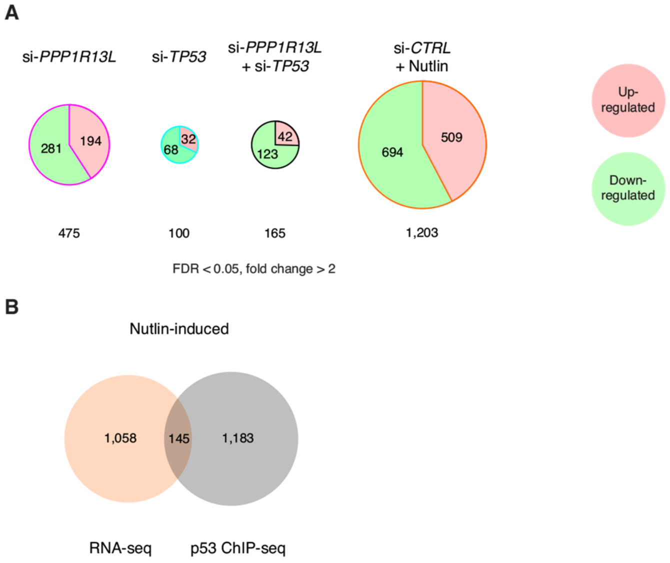

Fig. S1. Genomic analysis of p53–iASPP interactions. (*A*) Number of differentially [false discovery rate (FDR) < 0.05, fold change > 2] expressed genes compared to the control siRNA of each experimental condition established by RNA sequencing (RNA-seq). Numbers of up- and down-regulated genes in each sample are indicated. (*B*) Combining RNA-seq and p53 ChIP-seq identifies 145 differentially expressed genes with differential p53 binding (fold change > 2) in Nutlin-treated cells.

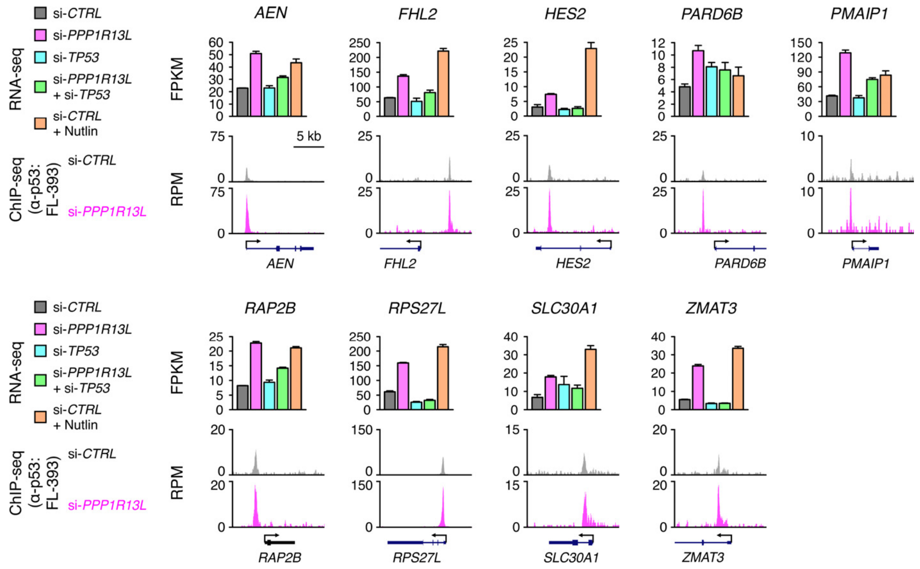

Fig. S2. iASPP-regulated p53 target genes. FPKM values from RNA-seq analysis of different cellular conditions (*Top*) and UCSC Genome Browser views of p53 occupancy from ChIP-seq (*Bottom*) for the remaining iASPP-regulated p53 target genes (iPTGs). UCSC Genome Browser views all have a 15-kb window (scale bar shown for *AEN*). Two batches of RNA-seq data ($n = 2$) were used. Error bars denote standard deviations.

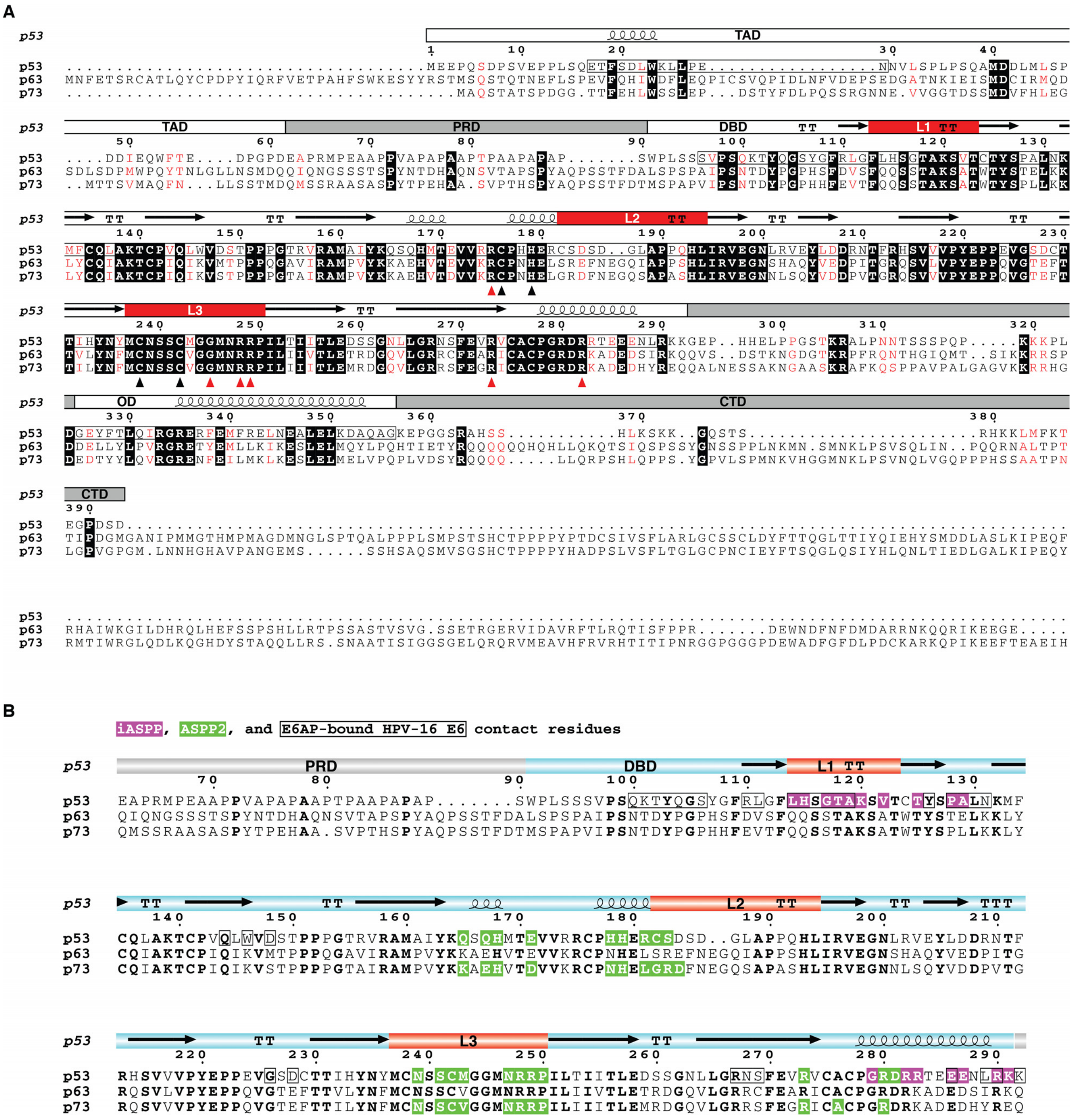

Fig. S3. Sequence alignments of the human p53 family. (*A*) The amino-acid sequences of human p53 (UniProt identifier P04637-1), p63 (UniProt identifier Q9H3D4-1), and p73 (UniProt identifier O15350-1) are aligned using Clustal Omega (https://www.ebi.ac.uk/Tools/msa/clustalo/). Identical residues among the family members are boxed in black, and similar residue types are colored in red. The modular architecture of p53, illustrated above the alignment, comprises an amino (N)-terminal transactivation domain (TAD), followed by a proline-rich domain (PRD), a central sequence-specific DNA-binding domain (DBD), an oligomerization domain (OD) that putatively mediates homo-tetramerization, and a carboxy (C)-terminal domain (CTD) that reportedly binds DNA non-specifically. Also shown above the alignment are the secondary structural elements annotated from representative published experimental structures of p53: an N-terminal sequence that folds into an α-helix recognized by MDM2 [residues 17–29; Protein Data Bank (PDB) accession code 1YCR chain B]; its central DBD from a complex with a cognate DNA sequence (residues 96–289; PDB accession code 1TUP chain B), and its OD (residues 325–356; PDB accession code 1C26). The so-termed L1, L2, and L3 loops critical for recognizing DNA are highlighted in red. The six cancer-derived mutational hot spots of p53 in the DBD are indicated below the alignment by red triangles and the four zinc-coordinating residues by black ones. (*B*) A close-up of the region corresponding to our p53 crystallization construct (residues 62–292). The PRD, which is not traceable in our electron density map, is greyed out while the DBD is colored in cyan. The secondary structural elements from our p53 model in complex with iASPP are annotated above the alignment. iASPP (from our structure) or ASPP2 [from p53 (PDB accession code 1YCS chain A) and p73 (PDB accession code 4A63 chain A) complexes] contact residues are boxed in magenta or green, respectively while E6AP-bound HPV-16 E6-binding residues (from PDB accession code 4XR8 as chain C) are framed. The distinct interaction interfaces for ASPP2 versus iASPP and E6AP-sculptured HPV-16 E6 are also evident in the amino-acid sequences.

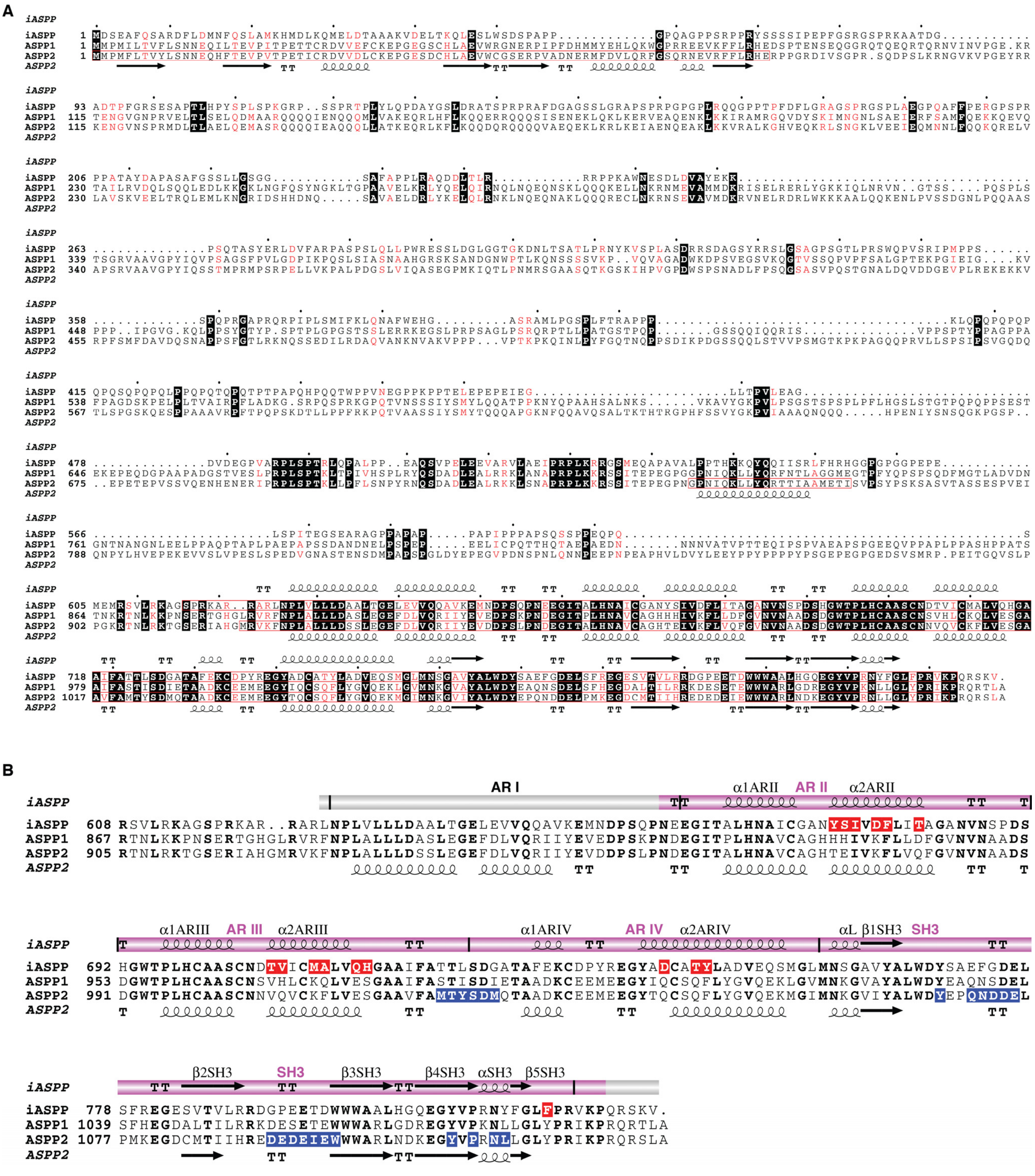

Fig. S4. Sequence alignments of human ASPP family members. (*A*) Human iASPP (UniProt identifier Q8WUF5-1), ASPP1 (UniProt identifier Q96KQ4-1), and ASPP2 (UniProt identifier Q13625-1) are aligned with secondary structural elements annotated from published experimental structures [iASPP: its C-terminal region alone (residues 616–823; PDB accession code 2VGE); ASPP2: its amino-terminal ubiquitin-like fold (residues 1–83; PDB accession code 2UWQ model 1), a central peptide sequence that folds into an α-helix mediating its interaction with *Helicobacter pylori* CagA oncoprotein (residues 746–765; PDB accession code 4IRV chain E), and its C-terminal region from a complex with p73 (residues 920–1121; PDB accession code 4A63 chain B)]. Identical amino-acid residues are boxed in black while similar residue types are colored in red. (*B*) The C-terminal conserved region of the family. The secondary structural elements from p53 complexes of iASPP (our structure) and ASPP2 (PDB accession code 1YCS chain B) are annotated above and below the alignment, respectively. In their C-terminal quarters, ASPP family members share the arrangement of an ankyrin repeat stack (AR I–IV) in intimate contact with a subsequent SH3 domain. Unexpectedly, AR I of iASPP appeared to be disordered in our complex with p53 and thus dispensable for engaging p53. The p53-binding residues of iASPP and ASPP2 are boxed in red and blue, respectively. iASPP and ASPP2 evidently employ distinct residues in homologous sequences to interact with p53.

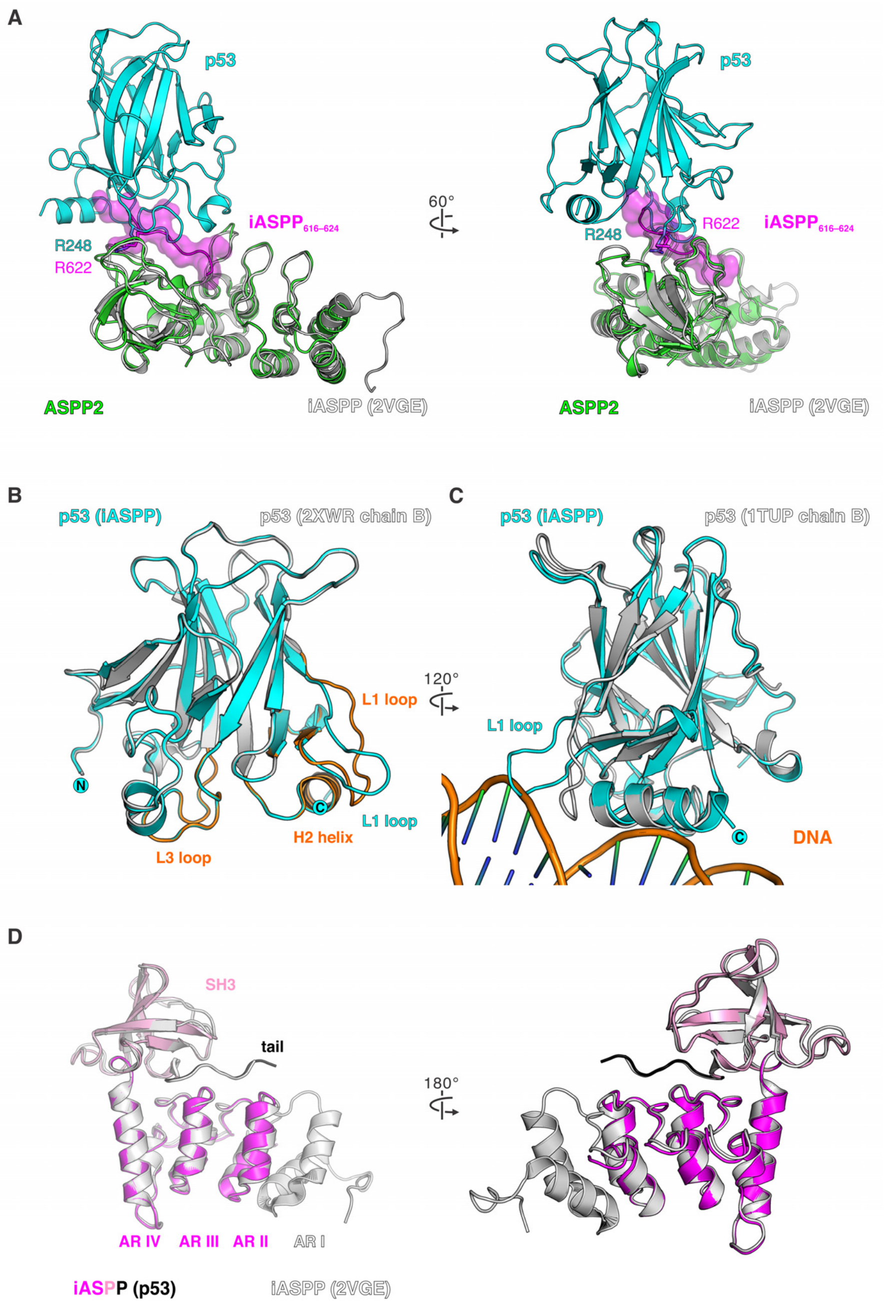

Fig. S5. Construct design and structures of the individual components in the p53–iASPP complex. (*A*) Design of iASPPcrys. The apo structure of iASPP (white; PDB accession code 2VGE] is superposed on ASPP2 (green) in its complex with p53 (cyan; PDB accession code 1YCS). The iASPP peptide (residues 616–624) immediately N-terminal to the ankyrin repeats from a symmetry-related molecule in the crystal lattice (PDB accession code 2VGE) is shown as magenta cartoon (over-laid with semi-transparent surface representation) with the side chain of R622 as sticks. The side chain of p53 R248 is also illustrated as sticks. (*B* and *C*) Comparison of the iASPP-complexed p53 DBD to apo (*B*) and DNA-complexed (*C*) p53 DBD structures. An apo structure of p53 DBD (*B*) (white; with an extended N terminus; PDB accession code 2XWR chain B; the search model used for molecular replacement) and a structure of the DNA (orange cartoon)-bound p53 DBD (*C*) (white; PDB accession code 1TUP chain B) are respectively super-posed on the observed structure of iASPP-complexed p53 (cyan). The major DNA-binding elements of p53, including the L1 and the L3 loops and the H2 helix, are colored in orange for the apo p53 DBD structure (*B*). (*D*) The published apo structure of iASPP (white; PDB accession code 2VGE; the search model used for molecular replacement) is superposed on the determined structure of p53-bound iASPP (colored as in Fig. 3*A*).

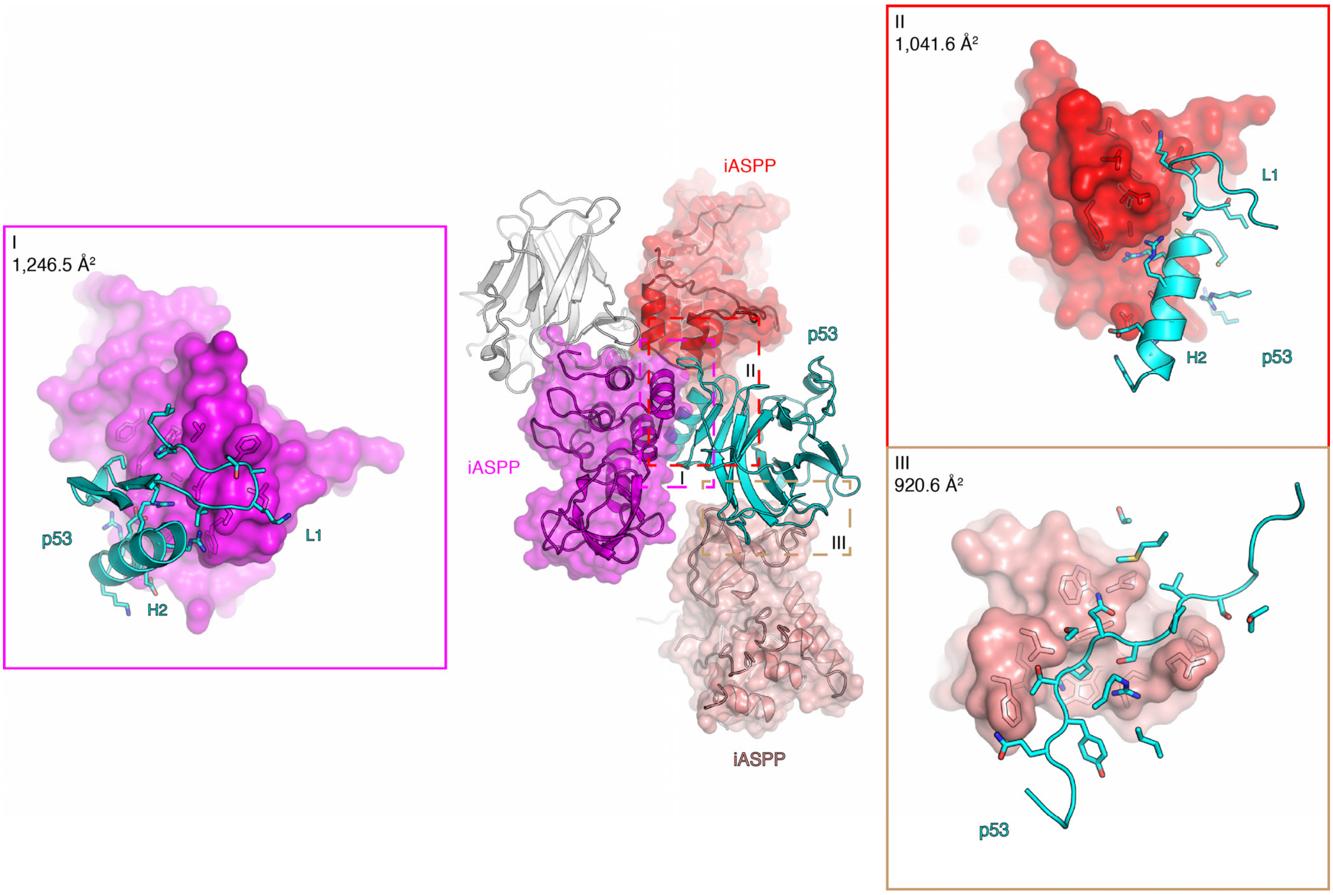

Fig. S6. The observed p53–iASPP interfaces. Three intermolecular interfaces between p53 and iASPP were observed in the crystal structure of our p53–iASPP complex. Interface I has the largest buried solvent-accessible surface area. At this interface, iASPP (magenta) predominantly uses a relatively flat face of its ankyrin stack (composed of residues exposed on the second α-helix in each ankyrin repeat) to contact the loop-sheet-helix motif of p53. Interface I has both hydrophobic and hydrophilic elements. Interface II is the second largest and is composed predominantly of hydrophilic interactions. At this interface, iASPP (red) uses a different face of its ankyrin stack to engage p53 at a surface that overlaps with its DNA-binding area. Interface III is the smallest of the three where iASPP (salmon) uses solely its SH3 domain to contact the N-terminal loop of p53 DBD, which wraps around the DBD, and the surrounding p53 residues.

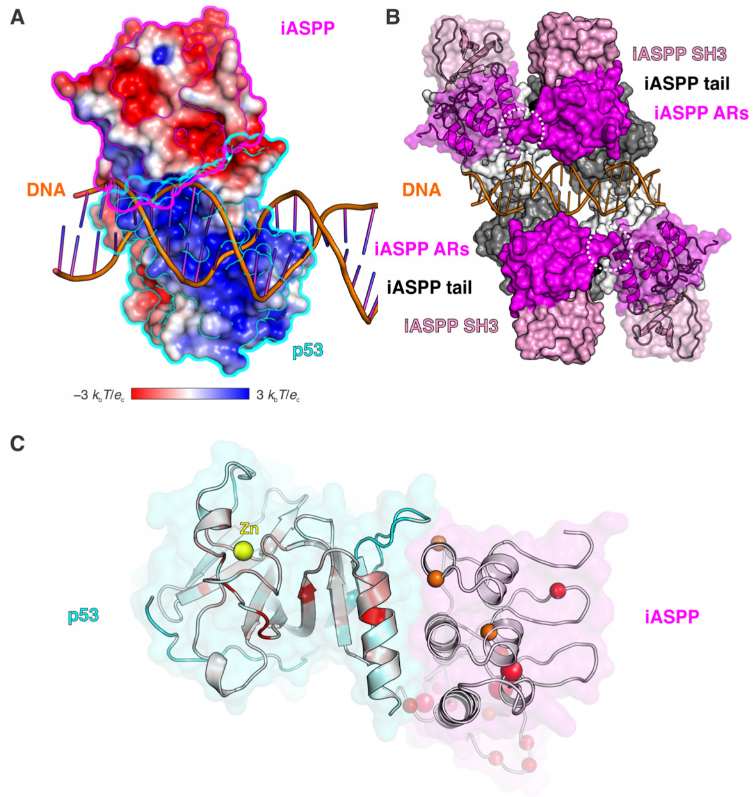

Fig. S7. DNA–p53–iASPP models and cancer-derived mutations. (*A*) Our iASPP complex is superposed on a DNA (orange cartoon) complex (PDB entry 1TUP) based on p53 and the solvent-accessible surface of our p53–iASPP complex is colored by electrostatic potential as indicated. p53 and iASPP are outlined in cyan and magenta, respectively. (*B*) A model of four p53–iASPP complexes respectively docked onto p53 DBD monomers in a DNA–tetrameric p53 complex (PDB entry 3KMD). The clashes between the ARs of the iASPP molecules related by the translational symmetry are indicated by dashed circles. (*C*) Cancer-derived p53 and iASPP mutations are mapped onto the crystal structure. Positions of iASPP mutations are shown as spheres: red indicates a tumor with wild-type *TP53* while orange indicates mutant *TP53*. The sizes of the spheres correlate with the number of cases and three positions have recurrent mutations. The relative mutational frequencies of p53 DBD are colored from cyan (rarely mutated) to red (frequently mutated). The p53-bound zinc^{2+} ion is shown as a yellow sphere.

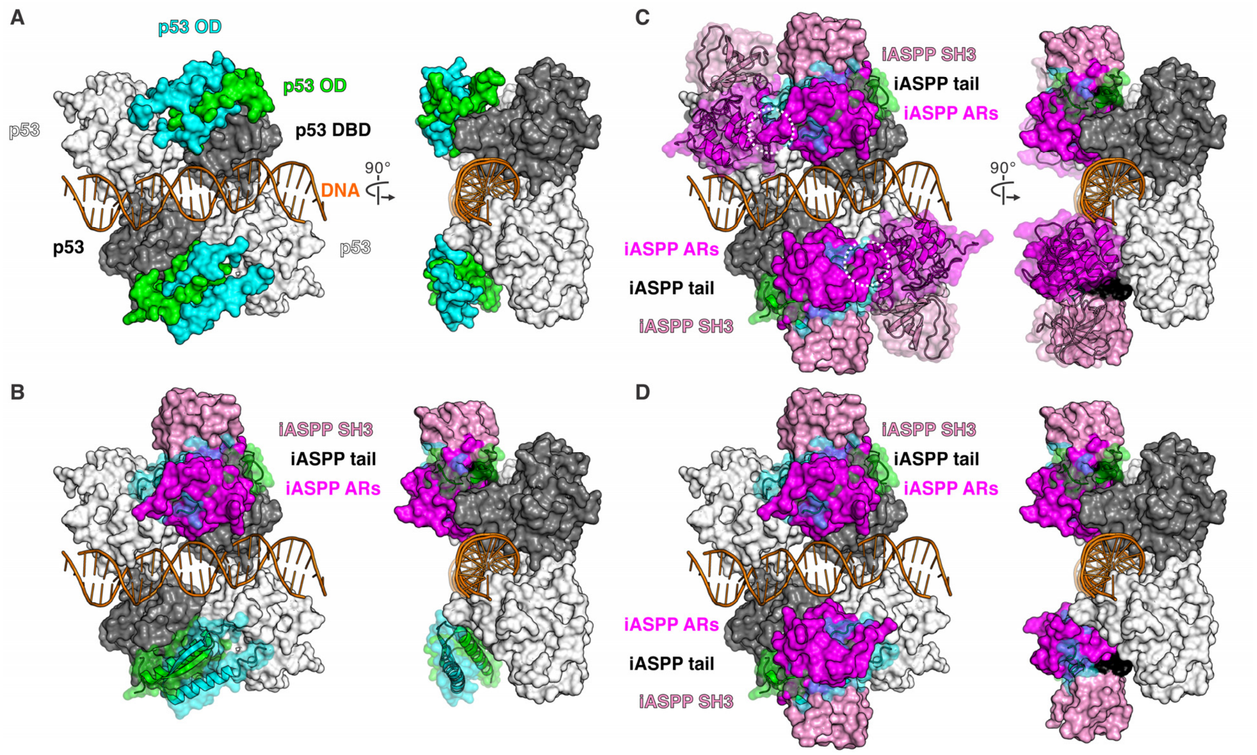

Fig. S8. An alternative model of the iASPP–p53–DNA assembly. (*A*) The prototypic *CDKN1A* response element (orange cartoon)-bound tetrameric p53 (surface representation) assembly (PDB entry 3TS8). The middle quarter sites-binding p53 DBD and the associated OD monomers are in grey and green, respectively, while the outer quarter sites-interacting DBD and the associated OD monomers are in white and cyan, respectively. (*B*) The iASPP complex superposed on the DNA–tetrameric p53 complex based on the superposition of the p53 DBD (one of the middle p53 DBDs as the reference). Note the steric clash between iASPP and the engineered, dimerized ODs. (*C*) A model of four p53–iASPP complexes respectively docked onto p53 DBD monomers in the DNA–tetrameric p53 complex. The clashes between the ARs of the iASPP molecules related by the translational symmetry are indicated by dashed circles. (*D*) A model of two iASPP molecules docked onto the middle two p53 DBD monomers in the DNA–tetrameric p53 complex.

Table S1. Crystallographic data collection and refinement statistics

**Data collection**	
Space group	$P4_12_12$
Cell dimensions	
a, *b*, *c* (Å)	71.1, 71.1, 255.6
α, *β*, *γ* (°)	90, 90, 90
Resolution (Å)	30.00–4.25 (4.40–4.25)
R_{merge}	0.158 (0.868)
$I/\sigma I$	8.3 (1.4)
Completeness (%)	93.1 (94.7)
Redundancy	3.9 (3.8)
**Refinement**	
Resolution (Å)	30.00–4.25
Number of reflections	4,819
R_{work}/R_{free}	0.243/0.292
Number of atoms	
Protein	2,866
Zn^{2+}	1
B-factors (Å^2)	
Protein	198.3
Zn^{2+}	210.6
Root-mean-square deviations	
Bond lengths (Å)	0.007
Bond angles (°)	1.156

The highest-resolution shell is shown in parentheses. 473 reflections were excluded from the refinement.

References

1. Martin M (2011) Cutadapt removes adapter sequences from high-throughput sequencing reads. *EMBnet.journal* 17(1):3.
2. Kim D, Langmead B, & Salzberg SL (2015) HISAT: a fast spliced aligner with low memory requirements. *Nat Methods* 12(4):357-360.
3. Trapnell C*, et al.* (2010) Transcript assembly and quantification by RNA-Seq reveals unannotated transcripts and isoform switching during cell differentiation. *Nat Biotechnol* 28(5):511-515.
4. Trapnell C*, et al.* (2013) Differential analysis of gene regulation at transcript resolution with RNA-seq. *Nat Biotechnol* 31(1):46-53.
5. Kuleshov MV*, et al.* (2016) Enrichr: a comprehensive gene set enrichment analysis web server 2016 update. *Nucleic Acids Res* 44(W1):W90-97.
6. Langmead B & Salzberg SL (2012) Fast gapped-read alignment with Bowtie 2. *Nat Methods* 9(4):357-359.
7. Zhang Y*, et al.* (2008) Model-based analysis of ChIP-Seq (MACS). *Genome Biol* 9(9):R137.
8. Heinz S*, et al.* (2010) Simple combinations of lineage-determining transcription factors prime cis-regulatory elements required for macrophage and B cell identities. *Mol Cell* 38(4):576-589.
9. Zeron-Medina J*, et al.* (2013) A polymorphic p53 response element in KIT ligand influences cancer risk and has undergone natural selection. *Cell* 155(2):410-422.
10. Wasserman WW & Sandelin A (2004) Applied bioinformatics for the identification of regulatory elements. *Nat Rev Genet* 5(4):276-287.
11. Walter TS*, et al.* (2005) A procedure for setting up high-throughput nanolitre crystallization experiments. Crystallization workflow for initial screening, automated storage, imaging and optimization. *Acta Crystallogr D Biol Crystallogr* 61(Pt 6):651-657.
12. Otwinowski Z & Minor W (1997) Processing of X-ray diffraction data collected in oscillation mode. *Methods Enzymol* 276:307-326.
13. McCoy AJ*, et al.* (2007) Phaser crystallographic software. *J Appl Crystallogr* 40(Pt 4):658-674.
14. Emsley P & Cowtan K (2004) Coot: model-building tools for molecular graphics. *Acta Crystallogr D Biol Crystallogr* 60(Pt 12 Pt 1):2126-2132.
15. Murshudov GN, Vagin AA, & Dodson EJ (1997) Refinement of macromolecular structures by the maximum-likelihood method. *Acta Crystallogr D Biol Crystallogr* 53(Pt 3):240-255.
16. Chen VB*, et al.* (2010) MolProbity: all-atom structure validation for macromolecular crystallography. *Acta Crystallogr D Biol Crystallogr* 66(Pt 1):12-21.
17. Chen S*, et al.* (2011) Structural and Functional Studies of LRP6 Ectodomain Reveal a Platform for Wnt Signaling. *Dev Cell* 21(5):848-861.
18. Krissinel E & Henrick K (2004) Secondary-structure matching (SSM), a new tool for fast protein structure alignment in three dimensions. *Acta Crystallogr D Biol Crystallogr* 60(Pt 12 Pt 1):2256-2268.
19. Krissinel E & Henrick K (2007) Inference of macromolecular assemblies from crystalline state. *J Mol Biol* 372(3):774-797.
20. Baker NA, Sept D, Joseph S, Holst MJ, & McCammon JA (2001) Electrostatics of nanosystems: application to microtubules and the ribosome. *Proc Natl Acad Sci U S A* 98(18):10037-10041.
21. Ashkenazy H, Erez E, Martz E, Pupko T, & Ben-Tal N (2010) ConSurf 2010: calculating evolutionary conservation in sequence and structure of proteins and nucleic acids. *Nucleic Acids Res* 38(Web Server issue):W529-533.
